# Quantum reservoir computing for photonic entanglement witnessing

**DOI:** 10.1126/sciadv.ady7987

**Published:** 2025-12-12

**Authors:** Danilo Zia, Luca Innocenti, Giorgio Minati, Salvatore Lorenzo, Alessia Suprano, Rosario Di Bartolo, Nicolò Spagnolo, Taira Giordani, Valeria Cimini, G. Massimo Palma, Alessandro Ferraro, Fabio Sciarrino, Mauro Paternostro

**Affiliations:** ^1^ Dipartimento di Fisica, Sapienza Università di Roma, P.le Aldo Moro 5, I-00185 Roma, Italy.; ^2^ Dipartimento di Fisica e Chimica Emilio Segrè, Università degli Studi di Palermo, via Archirafi 36, I-90123 Palermo, Italy.; ^3^ Quantum Technology Lab, Dipartimento di Fisica Aldo Pontremoli, Università degli Studi di Milano, I-20133 Milano, Italy.; ^4^ Centre for Quantum Materials and Technologies, School of Mathematics and Physics, Queen’s University Belfast, Belfast BT7 1NN, UK.

## Abstract

Accurately estimating properties of quantum states, such as entanglement, while essential for the development of quantum technologies, remains a challenging task. Standard approaches to property estimation rely on detailed modeling of the measurement apparatus and a priori assumptions on their working principles. Even small deviations can greatly affect reconstruction accuracy and prediction reliability. Here, we demonstrate that quantum reservoir computing embodies a powerful alternative for witnessing quantum entanglement and, more generally, estimating quantum features from experimental data. We leverage the orbital angular momentum of photon pairs as an ancillary degree of freedom to enable informationally complete single-setting measurements of their polarization. Our approach does not require fine-tuning or refined knowledge of the setup, at the same time outperforming conventional approaches. It automatically adapts to noise and imperfections while avoiding overfitting, ensuring robust reconstruction of entanglement witnesses and paving the way to the assessment of quantum features of experimental multiparty states.

## Teaser

A quantum reservoir uses photon OAM to witness entanglement without modeling, enabling robust, efficient, single-setting analysis.

## INTRODUCTION

The accurate estimation of quantum state properties remains a pivotal achievement for the advancement of quantum technologies. The golden standard in this context is embodied by quantum state tomography, whose goal is fully reconstructing a given quantum state (*1*, *2*), as well as quantum state estimation and certification protocols (*3*–*7*). More recently, classical shadows have shown how to efficiently estimate several properties of unknown quantum states, even for large state dimensions, provided suitable measurement strategies are used (*8*–*12*).

From an experimental perspective, we recognize two broad classes of measurement strategies. On the one hand, single-setting schemes (*13*–*18*) use a fixed experimental apparatus, thereby avoiding device reconfigurations. On the other hand, protocols based on multiple measurement bases—including standard tomographic approaches as well as strategies based on random measurements and shadow tomography (*19*–*24*)—can sometimes reduce resource requirements but necessitate frequent apparatus adjustments, intensifying the challenge of device calibration. Despite these operational differences, all such strategies hinge critically on an accurate modeling of both the dynamical evolution and the measurement stage: Unaccounted for features of the apparatus will inevitably lead to systematic errors and unreliable outcomes.

Several machine learning (ML) approaches have also been proposed to enhance the performance of state estimation protocols (*25*–*39*). ML-based methods, however, are often deficient in interpretability, and the opacity of the underlying models can complicate assessing the reliability of their results. These issues are further exacerbated when the goal is witnessing nonclassical features of quantum states, such as entanglement (*40*–*42*).

Here, we report the experimental realization of an estimation platform based on a memoryless variant of quantum reservoir computing, known as quantum extreme learning machines (QELMs) (*43*–*48*), intending to reconstruct entanglement witnesses on two-qubit entangled states encoded in the polarization degrees of freedom of photon pairs. More specifically, we use a double quantum walk (QW)–based apparatus in the polarization and orbital angular momentum (OAM) degrees of freedom (*49*, *50*) of two-photon input states. The QWs are implemented using passive and active optical elements, specifically polarization waveplates and q-plates (QPs) (*51*). This architecture allows embedding the polarization information into the much larger OAM space, whose measurement then yields an informationally complete measurement of the input polarization states (*52*). The choice of this architecture is motivated by its ability to embed information into the large OAM space using simple control devices such as polarization waveplates and QPs. This apparatus is not expected to be ideal for state reconstruction, since its effective measurement differs substantially from the symmetric measurements—such as mutually unbiased bases (MUBs) and SIC-POVMs—known to be optimal for such tasks (*8*, *9*). Moreover, accurately modeling all components of the apparatus and its noise sources is challenging. Nevertheless, as we will show, the proposed QELM strategy enables effective reconstruction even under such nonideal conditions. This provides a strong proof-of-principle demonstration that our strategy can enhance reconstruction performances across a broad range of experimental scenarios, extending far beyond the context of photonics.

Despite its memoryless nature, the resulting QELM protocol overcomes several limitations of existing approaches by offering key advantages. In particular, (i) it eliminates the need for prior modeling of the measurement apparatus, thereby completely circumventing the challenges associated with fine-tuning and calibration; (ii) its straightforward training procedure—amenable to rigorous theoretical analysis—ensures reliable and robust performances; (iii) by operating within a linear supervised ML framework, it sidesteps the interpretability and overfitting issues often encountered with more complex architectures.

Our QELM-based approach naturally aligns with the paradigm of self-calibrating strategies (*53*–*55*) by dynamically learning the optimal way to postprocess measurement data on-the-fly, thus mitigating the risk of miscalibration without extensive precalibration. Overall, the simplicity and generality of our approach make it adaptable to a wide range of experimental platforms and measurement devices and advantageous in any situation where characterizing the state preparation part of the experimental setup is easier than accurately modeling the whole evolution and measurement, including all sources of noise and misalignments in it. We furthermore show that our approach performs better than the standard tomographic method to reconstruct features of input states using the same hardware—which in this case consists of performing shadow tomography on the effective measurement describing the overall apparatus (*9*–*11*). The reason we decide to benchmark against the same hardware is that we wish to demonstrate the viability of our QELM strategy in general experimental measurement scenarios. To this end, one thus needs to directly compare the accuracy obtained using QELMs against alternative methods in each different experimental configuration.

To further highlight the capabilities and advantages of our formal approach, we demonstrate that models trained using only separable states are seamlessly capable of estimating entanglement features of previously unseen entangled states, achieving out-of-distribution generalization (*56*, *57*). This is in stark contrast to most ML-based approaches, which need a sufficiently representative training dataset to function. We report an innovative experimental implementation of a single-setting state estimation architecture that is model independent and allows to witness nonclassical features of input states. As such, our work marks a substantial step forward from previously reported reconstructions of single-qubit properties (*52*).

## RESULTS

### QELM-based entanglement witnessing

#### Overview of QELMs

QELMs offer a streamlined approach to quantum state estimation by leveraging uncharacterized but fixed quantum dynamics (*47*, *52*, *58*). In this framework, input states evolve through a quantum channel that acts as a “reservoir” by dispersing the information into a larger Hilbert space before measurement. A linear readout layer is then trained on a set of precharacterized states to recover the target features from the measurement data. In doing so, QELMs automatically learn the optimal mapping from measurement outcomes to desired features, thereby eliminating the need to fully characterize the quantum channel or measurement apparatus. Moreover, by restricting the postprocessing to linear operations, the method avoids overfitting when the target features are linear functions of the input density matrices—as is the case for the expectation value of any observable. This robustness arises from the fact that, quantum mechanically, output probabilities are inherently linear functions of the input states.

#### Formal description

Formally, a QELM is implemented via a “reservoir dynamic” comprising a quantum channel Φ and a positive operator–valued measure (POVM) $\mu \equiv {\left\{\mu_{b}\right\}}_{b=1}^{N_{\text{out}}}$, where $N_{\text{out}}$ denotes the number of measurement outcomes. The channel Φ describes the evolution of each input state, while the POVM describes how the output states are measured. The training objective is to determine a linear operator W that, when applied to the measurement statistics of an input state ρ, extracts its desired features. In the testing stage, the output probabilities $p_{b}\left(\rho \right)\equiv \text{Tr}\left[\mu_{b}\Phi \left(\rho \right)\right]$ are estimated from a finite statistical sample, yielding estimates ${\hat{p}}_{b}$, which are then combined linearly as $\sum_{b}W_{b}{\hat{p}}_{b}$ to produce the final output. This protocol does not require prior knowledge of Φ or μ; their effective action is automatically learned during training.

#### Training

The operator W is obtained via a supervised training procedure using a set of known training states ${\left\{\rho_{k}^{\text{tr}}\right\}}_{k=1}^{N_{\text{tr}}}$. For each training state, N copies are generated, evolved, and measured. The resulting data are organized into a probability matrix ${\hat{P}}_{N}$, which converges—in the N→∞ limit—to the exact probability matrix $P_{b,k}\equiv \text{Tr}\left[\mu_{b}\Phi \left(\rho_{k}^{tr}\right)\right]$. If the objective is to estimate the expectation value of an observable O, one solves for W the linear system

$$W{\hat{P}}_{N}=M_{O}$$ (1)

where $M_{O}$ is a row vector whose *k*th element is the expectation value $\text{Tr}\left(O\rho_{k}^{\text{tr}}\right)$. When training for multiple observables ${\left(O_{j}\right)}_{j=1}^{N_{\text{obs}}}$, $M_{O}$ becomes an $N_{\text{obs}}\times N_{\text{tr}}$ matrix with entries ${\left(M_{O}\right)}_{j,k}\equiv \text{Tr}\left(O_{j}\rho_{k}^{\text{tr}}\right)$ and W correspondingly a $N_{\text{obs}}\times N_{\text{out}}$ matrix. In this context, the training states are assumed to be known. In practice, this assumption amounts to assuming a high degree of control of the input state preparation stage of the experiment. Imperfections in state preparation will result in imperfections in the estimation. A straightforward solution to this linear regression problem is provided by the ordinary least squares estimator computed via the pseudoinverse

$$W=M_{O}{\hat{P}}_{N}^{T}{\left({\hat{P}}_{N}{\hat{P}}_{N}^{T}\right)}^{-1}$$ (2)

Alternative approaches, such as ridge regression or gradient descent–based methods, can be used when the system is ill conditioned—a scenario that may arise with many outcomes and high training statistics (*47*). In our experiments, the pseudoinverse method proved optimal, and we thus assume henceforth that W is computed via the pseudoinverse. The solvability of Eq. 1 is guaranteed provided that the effective POVM ${\tilde{\mu }}_{b}\equiv \Phi^{†}\left(\mu_{b}\right)$ is informationally complete, the training statistics are sufficiently large, and the training states span linearly the full state space (*47*). Under these conditions, the system can be solved for any target observable O, enabling the QELM to recover arbitrary information about the measured states. For further details on the training process, we refer to section S1 of the Supplementary Materials.

#### Connection with shadow tomography

QELMs are closely related to shadow tomography methods, whose core idea is that, given suitable measurement schemes, one can construct shadow estimators that accurately estimate target features with relatively few measurements (*8*, *9*). Effectively, training a QELM produces a shadow estimator tailored to the specific apparatus without requiring explicit knowledge of its internal workings: In the limit of large training statistics, the linear operator W converges to the estimator used in shadow tomography (*9*, *47*). In other words, QELMs exchange the need for a full characterization of the measurement apparatus—required in shadow tomography—for the simpler task of characterizing a restricted set of input states. This shift is particularly advantageous when accurately preparing the input states is easier than accurately modeling the entire experimental apparatus.

#### Optical implementation

In our experiment, the quantum channel is realized as an isometric evolution implemented via a two-photon QW in polarization and OAM. Each photon evolves independently through a QW apparatus composed of a sequence of polarization waveplates and QPs (*30*–*32*, *52*, *59*–*61*), more details can be found in Materials and Methods. This design embeds the information encoded in the polarization degree of freedom into the larger OAM space. After projecting the polarization onto a fixed direction, a projective measurement is performed over the OAM computational basis. In this configuration, the OAM serves as a reservoir—namely, an ancillary degree of freedom that enables the implementation of a single-setting, informationally complete measurement of the polarization degrees of freedom. Formally, the evolution of the initial state ρ is described by the completely positive map

$$\Phi \left(\rho \right)=\langle \eta_{p}|U\left(\rho ⊗|0\rangle \langle 0|\right)U^{†}|\eta_{p}\rangle$$ (3)

where $U\equiv U_{1}⊗U_{2}$ is the overall unitary evolution implemented by the apparatus, *U*_1_ and *U*_2_ correspond to the two independent QWs, $\eta_{p}$ is the projection onto the photon polarization, and ∣0〉 denotes the initial OAM state of the photons. To test the performance of the reservoir in different scenarios, we repeat the experiment with multiple QW configurations, by changing the rotation angles of the waveplates. Owing to imperfections and thermal fluctuations, the actual apparatus deviates from this idealized model. However, it is important to stress that this idealized model does not enter the training stage, meaning that the performance of the QELM remains completely agnostic to it.

#### Estimating entanglement witnesses

Once the QELM is trained on a complete set of states—that is, a set of density matrices spanning the entire state space—it can be used to estimate arbitrary observables for previously unseen quantum states. Crucially, even if this training set consists only of pure product states, the QELM can still capture entanglement features of unseen entangled states, as first remarked in (*62*). To demonstrate this capability, we use the QELM to estimate the expectation values of entanglement witnesses. An entanglement witness W is an observable whose expectation value is guaranteed to be nonnegative for all separable states but negative for at least one entangled state (*63*). Hence, measuring Tr(Wρ)<0 certifies that the state ρ is entangled. A standard way to construct entanglement witnesses is by defining the observable (*63*, *64*)

**Fig. 1.**
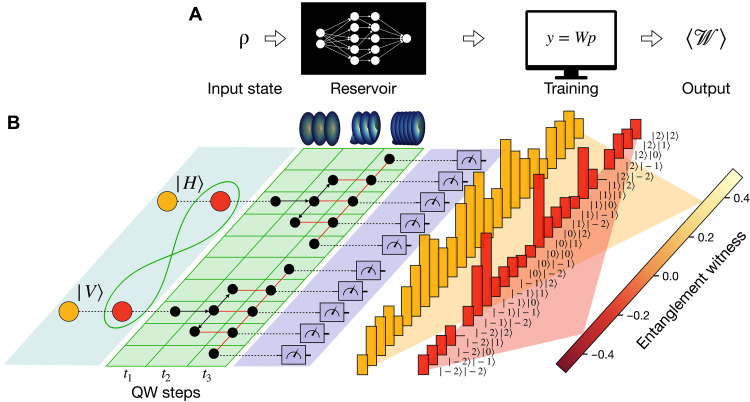
Schematic overview of the QELM experiment. (**A**) The protocol describes the evolution of an input state through a fixed and unknown quantum channel called reservoir, which maps the state to a higher dimensional Hilbert space. Here, through projective measurements in a single set configuration, the output probability distribution {p} of the state over the basis $\mu ={\left\{\mu_{b}\right\}}_{b=1}^{N_{\text{out}}}$ is retrieved. These probabilities are used in the final training stage to reconstruct the expectation value of an observable on the state. (**B**) In the experimental realization, entangled and separable quantum states encoded in the polarization degree of freedom of photon pairs evolve through the reservoir dynamics implemented by a double quantum walk configuration. Through the evolution, the 4-dimensional input space is enlarged into the 25-dimensional space of the OAM. By performing projective measurements on the computational basis of the latter, we obtain the output outcome probabilities on which the QELM model is trained to reconstruct a target entanglement witness.

W=αI−∣ψ〉〈ψ∣ (4)

where I is the identity operator, ∣ψ〉 is a target entangled state, and the parameter α is given as

$$\alpha =\underset{\rho \;\text{separable}}{\text{max}}\text{Tr}\left(\rho |\psi \rangle \langle \psi |\right)$$ (5)

thus representing the maximum overlap between the target state and separable ones. A depiction of the formal approach entailed by our QELM-based method is given in Fig. 1.

### Experimental implementation

#### Input state preparation

**Fig. 2.**
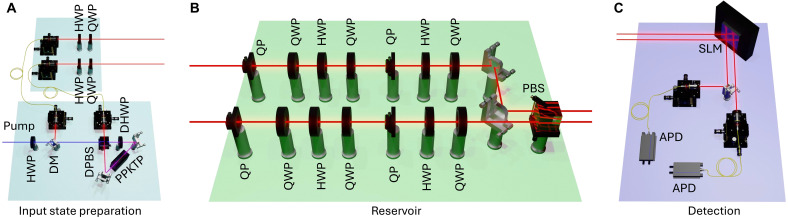
Experimental setup. (**A**) Input state preparation: Photon pairs are generated by spontaneous parametric down-conversion in a type II periodically poled potassium titanyl phosphate (PPKTP) crystal enclosed in a Sagnac interferometer. In the Sagnac interferometer, a dual-wavelength half-wave plate (DHWP) allows the nonlinear process to happen in each arm while compensating for the propagation delay acquired inside the crystal by the orthogonally polarized photons. The generated photons are separated by a dual-wavelength polarizing beam splitter (DPBS), while the pump laser is separated by a dichroic mirror (DM). After generation, each photon enters a layer consisting of a half-wave plate (HWP) and a quarter-wave plate (QWP) which encodes the input polarization state. (**B**) Reservoir evolution: After the state preparation, each photon enters an independent discrete-time QW consisting of a series of HWPs, QWPs, and QPs, which transfer the polarization information into the OAM degree of freedom. This QW implements the reservoir dynamics needed by the QELM. The polarization of the photons exiting the QW is then projected with a HWP, a QWP, and a polarizing beam splitter (PBS). (**C**) Detection: The final detection stage consists of a projective measurement in the OAM space, realized by a spatial light modulator (SLM) followed by coupling into single-mode fibers. This implements a projective measurement in the basis {∣n〉:n=−2,…,2}. Last, avalanche photodiode detectors (APDs) are used to collect the photons and detect the coincidence counts.

A schematic representation of the experimental apparatus is reported in Fig. 2. The input state preparation stage involves the spontaneous parametric down-conversion photon-pair source, with a periodically poled potassium titanyl phosphate crystal in a Sagnac configuration. This architecture enables the generation of stable polarization-entangled states. By adjusting the pump polarization, we can switch between generating separable and maximally entangled states. We will refer to these states as “reference states,” to distinguish them from the input states that are generated from them. The reference entangled state is in all cases $|\Psi^{+}\rangle =\frac{1}{\sqrt{2}}\left(|\mathrm{HV}\rangle +|VH\rangle \right)$. After the generation, the two photons are separated and sent to the input layer of the setup, where each one is manipulated using a half-wave plate (HWP) and a quarter-wave plate (QWP), with orientations chosen at random and defining the input polarization states entering the reservoir. Overall, by rotating also the waveplate in the pump beam, two distinct sets of random two-qubit polarization states for training and testing the QELM are generated, one consisting of product states and the other of maximally entangled states.

#### Reservoir dynamic and measurement

Once generated, the two-photon states are fed into the core computational layer, where the reservoir dynamics are realized by two parallel QW evolutions on both polarization and OAM degrees of freedom of the photons (see Fig. 2 and Materials and Methods for further details on the experimental implementation) (*59*, *60*). The resulting double QW evolution generates output states spanning a 25-dimensional walker space, given by the OAM values {∣n〉,n=−2,...,2} for each photon. After the reservoir evolution, each photon’s polarization is projected using a combination of waveplates and a polarizing beam splitter. Next, a spatial light modulator combined with the coupling to single-mode fibers performs projective measurements in the two-photon OAM space. Last, the photons are detected by avalanche photodiode detectors. Thanks to the dimensionality increase induced by the QWs, this final projective OAM measurement results in a nonprojective, single-setting, informationally complete POVM on the two-photon input. Repeating this procedure multiple times for each input state, we obtain the dataset used to train and test the QELM.

#### Summary of experimental settings

To benchmark the performance of our platform, we performed experiments under three configurations, labeled E1, E2, and E3. Each configuration is characterized by different QWP and HWP angles specifying the reservoir’s unitary and by the method used to prepare the input states. Specifically:

1) E1 uses waveplate angles that were numerically optimized to minimize the average reconstruction mean-squared error (MSE), similarly to how is done in (*52*).

2) E2 is derived from E1 by swapping two of the optimized angles, perturbing the reservoir dynamics.

3) E3 uses a fully random set of waveplate angles, sampled uniformly.

These choices were made to benchmark the hardware performance across ideal (E1), perturbed (E2), and “random” (E3) scenarios. The reference separable state—that is, the photons state that exits the photon generation and enters the state preparation layer when using separable states—is $|\Psi_{R}\rangle =|\mathrm{VV}\rangle$ for E1 and $|\Psi_{R}\rangle =|\mathrm{VH}\rangle$ for E2 and E3. These choices are made to test the robustness of the configuration under different conditions, input states, and reservoir dynamics. In all cases, the same angles are used in both QWs, so that every injected state has the form $\left(U_{\text{in}}⊗U_{\text{in}}\right)|\Psi_{R}\rangle$, with $U_{\text{in}}$ the single-photon reservoir evolution. In each configuration, we test an equal number of separable and entangled states, namely, 150 for E1 and E3 and 83 for E2. This difference in the statistics used in E1 versus E2 and E3 is simply due to experimental issues during the data acquisition process; we expect this difference to bear no significant impact on the reported results, and we only report it for completeness. The preparation angles are sampled uniformly at random in all cases. Additional experimental details can be found in sections S2 and S3 of the Supplementary Materials.

#### Entanglement witness performance with mixed training

**Fig. 3.**
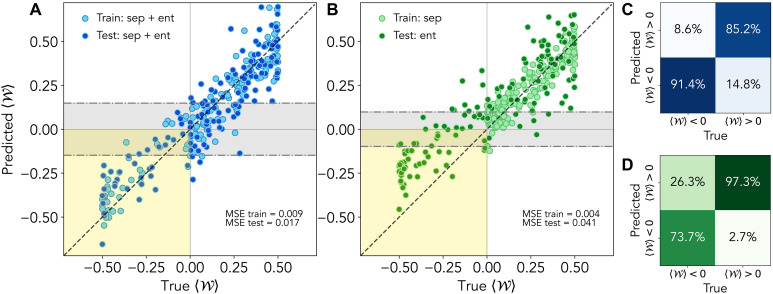
Performance of entanglement witness estimation. Predicted versus true values of the witness W for the E1 scenario under different training configurations. In (**A**), both training and test datasets contain separable and entangled states, while in (**B**), the training set includes only separable states and the test set only entangled ones. These results showcase how the model’s linearity allows it to train on only states with W>0 and still accurately predict previously unseen states with W<0 (yellow area in the plot). The shaded gray region in each plot represents the estimation error, defined as the square root of the training MSE. Last, (**C**) and (**D**) report the confusion matrices showing the accuracy in correctly identifying positive and negative values of W, corresponding to the data in (A) and (B), respectively.

We consider here the task of reconstructing the expectation value of the entanglement witness $W\equiv \frac{1}{2}\left(I-2|\Phi^{+}\rangle \langle \Phi^{+}|\right)$, with $|\Phi^{+}\rangle \equiv \frac{1}{\sqrt{2}}\left(|\mathrm{HH}\rangle +|\mathrm{VV}\rangle \right)$, when training the QELM with both separable and entangled states. Notably, our protocol works independently of the choice of the specific witness as long as the corresponding operator is linear with respect to the input state (*47*). To assess reconstruction performances, we report the MSE on both training and test states. The MSE is here defined as the average squared difference between predicted and true 〈W〉, for each state. This directly quantifies the absolute estimation error, which is particularly relevant in our setting where the dominant contribution to the total error arises from stochastic noise that is independent of the magnitude of the observable being estimated. Figure 3 reports the results for the E1 configuration, in which training and testing use two sets, each comprising 150 separable and 150 entangled states. Figure 3A demonstrates a strong agreement between reconstructed and true expectation values. The MSEs averaged over the training and test states are ${\text{MSE}}_{\text{train}}≃0.009$ for training and ${\text{MSE}}_{\text{test}}≃0.017$ for testing. From the perspective of certifying entanglement, we quantify the apparatus accuracy as the fraction of entangled states with 〈W〉<0, for which the model also correctly predicts 〈W〉<0. These correspond to the points in the shaded lower-left region in Fig. 3A. As shown in the confusion matrix in Fig. 3C, entanglement is correctly certified for 91.4% of these states. These estimates are subject to stochastic errors arising, among other factors, from finite sampling statistics. It is therefore sensible to consider a state as reliably certified as entangled only if the estimated witness value is more negative than a threshold determined by the training MSE. Taking this into account—and using a threshold of three SDs, where the SD is computed as the square root of the training MSE—we find that 37.1% of entangled states remain certifiably entangled even within this elevated error tolerance. These numbers depend weakly on the specific subset of random training states chosen. Averaging these errors over random training instances we get ${\bar{\text{MSE}}}_{\text{train}}=0.010\pm 0.001$ and ${\bar{\text{MSE}}}_{\text{test}}=0.015\pm 0.001$, with a number of certified entangled states equal to (89±4)%. The reported errors represent one SD. These results showcase the good estimation performances of the QELM strategy even in relatively noisy conditions and without the protocol requiring knowledge of the underlying dynamic.

#### Generalization performances

Another notable consequence of QELMs restricting to linear postprocessing is the model’s amenability to domain generalization, particularly in the context of entanglement witnessing. Because training a QELM enables it to learn how to estimate target observables, and because separable states alone span the space of all states, it follows that a QELM can be trained exclusively on separable states yet still perform well on previously unseen entangled states. To demonstrate this, we present in Fig. 3B the results obtained when the training dataset contains all 150 separable states, while the test dataset contains all 150 entangled ones. We again observe strong agreement between the true and predicted values of 〈W〉 and that many states are reliably identified as entangled—although the model was never trained on any state corresponding to negative 〈W〉. The training and test MSEs are ${\text{MSE}}_{\text{train}}≃0.004$ and ${\text{MSE}}_{\text{test}}≃0.04$. The fraction of correctly certified entangled states, as reported in Fig. 3D, is 73.7%. Accounting for stochastic errors, 32.9% of entangled states remain certified within three SDs.

#### Effect of input state mislabeling

**Fig. 4.**
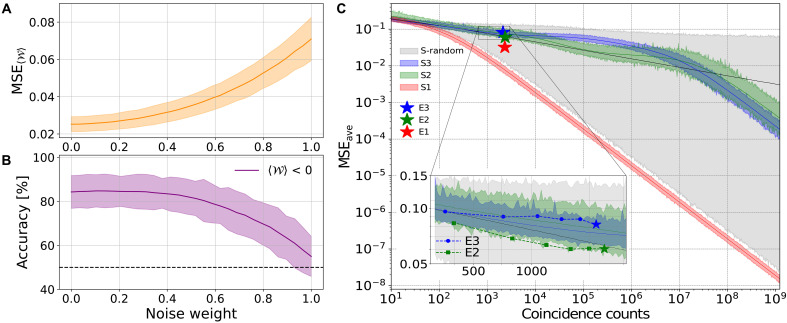
Dependence of estimation performances on noise and choice of reservoir. (**A**) and (**B**) report the robustness of the witness estimate 〈W〉 under noise, where the entangled reference state $\rho_{\text{ent}}$ is replaced by statistical mixtures of the form $\rho_{\text{noise}}=\left(1-p\right)\rho_{\text{ent}}+p\rho_{\text{sep}}$, where p∈[0,1] is the noise parameter. Here, the studies are performed in the experimental scenario E2. This scenario is not the optimal one but is obtained for a perturbed reservoir dynamic, swapping the QWP angles. In (A), the solid curve shows the average MSE on the test set as a function of p, and the shaded regions represent the SDs obtained by repeating the procedure with different random training instances. Each instance contains 150 training states and 150 test states. (B) reports the corresponding accuracy in identifying negative values of the 〈W〉, and the dotted line indicates the 50% accuracy value. (**C**) compares experimental performances (E1, E2, and E3) with numerical simulations (S1, S2, and S3). Stars indicate the experimental MSEs, while solid lines represent the corresponding numerical results under the same conditions and color coding. In all cases, training is performed on separable states and testing on entangled states; the MSE is averaged over the possible target observables and plotted against the collected statistics (fixed as the same in both training and test). The shaded regions around each simulation curve indicate one SD across sampling instances, while the boundaries of the gray shaded region mark the minimum and maximum averaged MSE obtained when training is performed with random reservoir configurations (S-random). The inset details the behavior of the experimental results for E2 and E3, showing the consistency between numerical simulations and experimental data across varying statistics.

To evaluate the noise resilience of the model, we constructed a new dataset of nonmaximally entangled states by taking a weighted combination of the experimentally measured OAM distributions for entangled and separable states. This amounts to using as reference states for the entangled system statistical mixtures of the form $\rho_{\text{noise}}=\left(1-p\right)\rho_{\text{ent}}+p\rho_{\text{sep}}$, with p a noise parameter, $\rho_{\text{ent}}$ the reference entangled state, and $\rho_{\text{sep}}$ the reference separable state. In any case, each of these mixed states was labeled with the expected value computed for the maximally entangled state. The study was performed in the experimental scenario E2, and Fig. 4 (A and B) illustrates the impact of noise on the model’s performance. In particular, the average MSE and the accuracy in identifying negative entanglement witness values as functions of p are reported, respectively, in the two panels. These results indicate that the model is highly resilient to noise, with performance remaining robust for noise levels up to p=0.5.

#### Dependence of performances on reservoir configuration

In Fig. 4C, we compare the performance of the three configurations in the numerical simulations and the experimental implementations. The data illustrate that different reservoir configurations can yield different estimation accuracies under the same training statistics. This is due to different configurations corresponding to different effective POVMs in the Heisenberg picture and thus naturally different estimation performances (*47*). Although our double-QW apparatus uses a relatively simple evolution—far from the ideal random unitary scenario associated with optimal performance—we still observe, at least in numerical simulations, the expected inverse proportionality between the average MSE and the number of collected samples N. This emerges after an initial settling regime in which the test statistics cannot be fully exploited due to insufficient training statistics: Until the estimator W is sufficiently unbiased, the MSE decreases at a slower rate than 1/N. For sufficiently large training statistics, we recover the expected behavior where the test MSE decreases as β/N, with β a proportionality constant that depends on the specifics of the measurement setting. Different reservoir configurations spread the encoded information among outcomes to varying degrees, which in turn influences how readily that information can be retrieved. As a result, certain choices of angles—in particular, those used in the E1 configuration—are sufficient to reach the 1/N regime with the available experimental statistics. In contrast, the configurations E2 and E3 require additional statistics before reaching the same performance level. In principle, this regime could be achieved experimentally by increasing the data acquisition time and, therefore, the number of coincidence counts for each state. For practical reasons, we have set the acquistion time to complete a full data acquisition within 10 hours having an average overall signal of ~3000 coincidence counts per state. Note that the apparently larger discrepancy between E1 experimental and simulated data is an artifact of the log-log scale: The discrepancy is in all cases found to have the same order of magnitude of ~10^−2^.

#### Benchmark with shadow tomography

To benchmark the performance of our QELM-based approach, we compare it against shadow tomography (*5*, *8*, *9*, *12*), a general methodology for computing the optimal unbiased estimator of target observables—that is, the unbiased estimator with the smallest average variance—in fixed-measurement scenarios. Because both approaches aim to efficiently extract properties of input states without requiring a full tomographic reconstruction, shadow tomography is the natural benchmark for our apparatus. For reservoir configuration E1, and the same task as Fig. 3, shadow tomography yields an MSE of approximately 0.015 for separable states and 0.072 for entangled states. In contrast, our QELM, trained solely on separable states, reduces these MSEs to 0.002 and 0.041, respectively. Doing the training on both separable and entangled states gives the even smaller MSEs of 0.009 and 0.017. Moreover, the reported shadow tomography MSEs represent lower bounds, since shadow tomography requires knowledge of the input statistics N, which is unavailable here. These findings clearly highlight the advantages of using a QELM-based approach for state property estimation. In principle, one could build a much more detailed physical model of the apparatus and, with it, push the shadow tomography performance beyond that of the QELM. In practice, however, constructing and validating such a model are very time consuming, resource intensive, and very susceptible to the specifics of the hardware. By contrast, our model-agnostic QELM pipeline avoids this overhead: It learns directly from experimental data and therefore adapts naturally to modifications in the setup while still delivering strong performance. For further details on this analysis, we refer to section S4 of the Supplementary Materials.

## DISCUSSION

We have experimentally demonstrated the capability of quantum reservoir computing, specifically in the QELM form, to reconstruct an entanglement witness for bipartite states of a two-photon system, achieving performance on par with methods that rely on careful calibration and prior knowledge of the apparatus. Crucially, our approach is platform independent: The QELM paradigm can be straightforwardly adapted to different experimental settings, thanks to its ability to adapt to measurement outcomes on-the-fly without explicit modeling of the underlying dynamics.

In particular, our photonic implementation leverages the OAM degree of freedom to realize a compact, single-setting measurement scheme that bypasses many practical issues of conventional multisetting protocols, such as the need for frequent device reconfiguration or stringent calibration requirements. As the QELM training procedure reduces to linear regression, we also circumvent interpretability challenges and overfitting issues commonly encountered in more complex ML architectures.

A key advantage of our method is its robustness: By demonstrating that training solely on separable states is sufficient to accurately witness the entanglement of previously unseen input states, we highlight how the QELM effectively self-calibrates to the experiment. Moreover, our findings reveal that drifts in the experimental setup can leave the final witness reconstruction largely unaffected, thus highlighting the resilience of such a technique.

Looking ahead, the QELM principles deployed here can be applied to more general quantum state estimation scenarios, paving the way for improved property estimation tasks in complex photonic platforms and beyond. Equally remarkable is the prospect of transfer learning demonstrations, where QELMs trained on fully classical data (e.g., coherent states) can later be applied to certify nonclassical properties of unknown quantum states, with no additional overhead. In virtue of its simplicity, flexibility, and generality, this method has thus the potential to become a valuable tool for the characterization of quantum technologies.

## MATERIALS AND METHODS

### QWs in the angular momentum

The QW evolution in the angular momentum of single photons is realized by encoding the coin and walker states, respectively, in their polarization and OAM degrees of freedom (*60*). Each QW step consists of a rotation of the internal coin state, implemented by three waveplates, followed by a controlled shift on the walker position, realized by the QP. The latter is a device made of a birefringent material designed to have a nonuniform pattern for its optical axis, which is engineered to enable the conditional change of the light OAM in function of its polarization (*51*). Formally, each coin operation is given by C(ζ,θ,ϕ)=QWP(ϕ)HWP(θ)QWP(ζ), where {ζ,θ,ϕ} are the waveplate rotation angles; the controlled shift operator S(α,δ) instead depends on the tuning parameter δ, which governs the coupling efficiency of the QP, and α, the initial angle of the device optical axis with respect to the horizontal one. Explicitly, using the circular polarization basis for the polarization, we have that

$$C\left(\zeta ,\theta ,\phi \right)=\left(\begin{array}{cc} e^{-i\left(\zeta -\phi \right)}\text{cos}\eta & e^{i\left(\zeta +\phi \right)}\text{sin}\eta \\ -e^{-i\left(\zeta +\phi \right)}\text{sin}\eta & e^{i\left(\zeta -\phi \right)}\text{cos}\eta \end{array}\right)$$ (6)

$$\begin{array}{cc} S\left(\alpha ,\delta \right)= & \sum_{n=-N+1}^{N-1}\text{cos}\frac{\delta }{2}\left(|L,n\rangle \langle L,n|+|R,n\rangle \langle R,n|\right)\\ & +i\text{sin}\frac{\delta }{2}\left(e^{2\text{iα}}|L,n\rangle \langle R,n+1|+h.c.\right) \end{array}$$ (7)

where η=ζ−2θ+ϕ, and ∣R〉(∣L〉) and ∣n〉 denote the right (left) circular polarization state and the OAM eigenstates, respectively. Adjusting δ allows partial controlled shift operations, which serves to further enlarge the accessible OAM space by using the unchanged component of the OAM (*52*). In particular, we implement a two-step evolution in each of the two QWs, so that the overall unitary is $U=U_{1}⊗U_{2}$, with

$$U_{k}=S\left(\alpha_{2}^{k},\pi \right)C\left(\zeta^{k},\theta^{k},\phi^{k}\right)S\left(\alpha_{1}^{k},\pi /2\right),k\in \left\{1,2\right\}$$ (8)

The parameters $\alpha_{1}^{1}=19{}^{\circ}$, $\alpha_{2}^{1}=77{}^{\circ}$, $\alpha_{1}^{2}=336{}^{\circ}$, and $\alpha_{2}^{2}=163{}^{\circ}$ are fixed by the QP fabrication process. After the evolution through this apparatus, each photon is in a superposition of the five OAM states {∣−2〉,∣−1〉,∣0〉,∣1〉,∣2〉}, leading to a total 25-dimensional output walker space.

## Supplementary Materials

### This PDF file includes:

Sections S1 to S4 Figs. S1 to S8 Tables S1 to S3
