# Supplementary material for: Quantum reservoir computing for photonic entanglement witnessing
Source: Sci Adv. 2025 Dec 12;11(50):eady7987. doi: 10.1126/sciadv.ady7987 (PMC12700208; doi:10.1126/sciadv.ady7987)
Supplement: Supplementary file 1 — Sections S1 to S4 Figs. S1 to S8 Tables S1 to S3 [file sciadv.ady7987_sm.pdf]

Science Advances
AAAS

Supplementary Materials for

Quantum reservoir computing for photonic entanglement witnessing

Danilo Zia *et al.*

Corresponding author: Fabio Sciarrino, fabio.sciarrino@uniroma1.it;
Mauro Paternostro, mauro.paternostro@unipa.it

Sci. Adv. **11**, eady7987 (2025)
DOI: 10.1126/sciadv.ady7987

This PDF file includes:

Sections S1 to S4
Figs. S1 to S8
Tables S1 to S3

S1. THEORETICAL BACKGROUND

General recipe — Building on a quantum reservoir-based architecture, a QELM uses a training dataset of *a priori* known states, each evolving under an unknown "reservoir dynamic", to learn how best to extract information from measurement data on new, previously unseen states. We can always model the reservoir dynamic as a quantum channel Φ followed by a POVM $\boldsymbol{\mu} \equiv \{\mu_1, \dots, \mu_{N_{\rm out}}\}$, with $N_{\rm out}$ the number of measurement outcomes. The training dataset comprises pairs $\{(\rho_i^{\rm tr}, o_i)\}_{i=1}^{N_{\rm tr}}$, where $\rho_i^{\rm tr}$ is the i-th training state and o_i is the target value the QELM should output for $\rho_i^{\rm tr}$. Alternatively, one can consider as training dataset the pairs $\{(f_i^{\rm tr}, o_i)\}_{i=1}^{N_{\rm tr}}$, with $f_i^{\rm tr}$ the set of frequencies obtained measuring $\rho_i^{\rm tr}$ a set number of times. Unless otherwise specified, o_i is taken to be an expectation value of one or more observables $\mathcal{O}^{(j)}$. Concretely, either $o_i = {\rm Tr}(\rho_i^{\rm tr}\,\mathcal{O})$ for some single observable $\mathcal{O}$, or $\boldsymbol{o}_i = \left({\rm Tr}(\rho_i^{\rm tr}\,\mathcal{O}^{(j)})\right)_{j=1}^{N_{\rm obs}}$ for $N_{\rm obs} > 1$ observables. Ultimately, the QELM is trained to produce a linear function W, which is then applied to measurement data from any — possibly previously unseen — input state to recover the desired expectation values. In this work, the target observable is taken to be either a tensor product of some pair of Pauli matrices, or an entanglement witness of the form $\mathcal{W}_{\Psi_i^{\rm Bell}} = \frac{I}{2} - |\Psi_i^{\rm Bell}\rangle\langle\Psi_i^{\rm Bell}|$, where $|\Psi_i^{\rm Bell}\rangle \in \{\,|\Phi^\pm\rangle\,, |\Psi^\pm\rangle\}$ are the four canonical Bell states. For brevity we will refer to these four witnesses as $\mathcal{W}_i$, $i = 1, 2, 3, 4$.

Advantages over alternative methods — Restricting a QELM to linear post-processing provides several advantages compared to more general machine-learning approaches to quantum state estimation [25-39]. In particular, training is simpler, the model is more interpretable, and the risk of overfitting is significantly reduced. The key reason is the linearity between input density matrices and outcome probabilities, valid in any quantum system regardless of Φ and $\boldsymbol{\mu}$. This linearity implies that, if the overall measurement is informationally complete, one can recover the input state from outcome probabilities via a linear function — which is precisely how QELMs operate [47]. If Φ and $\boldsymbol{\mu}$ were fully characterized, one could use standard shadow tomography [9] or full state tomography to estimate observables or reconstruct input states. However, these techniques rely on a precise model of the experimental apparatus. Any inaccuracy in this modeling degrades performance. In contrast, QELMs do not require explicit knowledge of Φ or $\boldsymbol{\mu}$, instead relying on a pre-characterized (training) set of input states. This can be advantageous because accurately preparing and modeling a subset of states is often much simpler than modeling the entire apparatus [52].

Effective POVM description — A convenient way to analyze a QELM is to treat the channel Φ and measurement $\boldsymbol{\mu}$ as a single *effective POVM* $\tilde{\boldsymbol{\mu}} \equiv \{\tilde{\mu}_1, \dots, \tilde{\mu}_{N_{\rm out}}\}$ acting directly on the input states in the Heisenberg picture, where $\tilde{\mu}_b = \Phi^\dagger(\mu_b)$, and $\Phi^\dagger$ is the adjoint of Φ. The outcome probabilities then simply read $p_b(\rho) = {\rm Tr}(\tilde{\mu}_b\,\rho)$. From the standpoint of extracting features of the input state, this $\tilde{\boldsymbol{\mu}}$ is the object that more directly affects performances, rather than Φ and $\boldsymbol{\mu}$ individually.

Training method — A defining feature of QELMs is their restriction to a *linear* model, meaning measurement data is only post-processed through linear (or affine) operations. Once trained, applying a QELM to a new input state ρ involves (1) measuring a number of copies of ρ to obtain a frequency vector $\boldsymbol{f}(\rho) \in \mathbb{R}^{N_{\rm out}}$ that approximates the true probabilities $\boldsymbol{p}(\rho) = \langle \tilde{\boldsymbol{\mu}}, \rho\rangle$, and (2) computing $\hat{o} = W\,\boldsymbol{p}(\rho)$, where W is the matrix obtained from the training. Finding W involves solving the linear system $W\langle\tilde{\boldsymbol{\mu}}, \boldsymbol{\rho}^{\rm tr}\rangle = \langle \boldsymbol{\mathcal{O}}, \boldsymbol{\rho}^{\rm tr}\rangle$, where the shorthand $\langle\tilde{\boldsymbol{\mu}}, \boldsymbol{\rho}^{\rm tr}\rangle$ denotes the $N_{\rm out} \times N_{\rm tr}$ matrix whose j-th column is the outcome probability vector for the j-th training state, and $\langle \boldsymbol{\mathcal{O}}, \boldsymbol{\rho}^{\rm tr}\rangle$ is the $N_{\rm obs} \times N_{\rm tr}$ matrix containing the corresponding true expectation values of the target observables. A canonical solution is $W = \langle \boldsymbol{\mathcal{O}}, \boldsymbol{\rho}^{\rm tr}\rangle\langle\tilde{\boldsymbol{\mu}}, \boldsymbol{\rho}^{\rm tr}\rangle^+$, where $(\bullet)^+$ denotes the Moore–Penrose pseudoinverse. This solution is guaranteed when $N_{\rm out} \leq N_{\rm tr}$ and $\langle\tilde{\boldsymbol{\mu}}, \boldsymbol{\rho}^{\rm tr}\rangle$ is surjective, which corresponds to the effective measurement being informationally complete and there being sufficiently many training states. When the number of training states is *strictly* larger than the number of outcomes, $N_{\rm out} < N_{\rm tr}$, there are multiple possible solutions for W. In such cases, the pseudoinverse corresponds to the solution minimizing the Euclidean norm.

Interpretability of the training process — As shown in [47], the canonical solution for W can be written as $W = \langle \boldsymbol{\mathcal{O}}, \tilde{\boldsymbol{\mu}}^\star\rangle$, that is, $W_{ib} = {\rm Tr}(\mathcal{O}_i\,\tilde{\mu}_b^\star)$, with $\tilde{\mu}_b^\star = S^{-1}(\tilde{\mu}_b)$ the so-called *canonical dual POVM*, and S the quantum map with action $S(X) = \sum_b \tilde{\mu}_b\,{\rm Tr}(\tilde{\mu}_b\,X)$ [9, 47]. Thus, training a QELM effectively recovers the dual of the underlying (effective) measurement.

Effects of finite statistics on the training — The relation between W and $\tilde{\boldsymbol{\mu}}^\star$ holds exactly only when using the exact probabilities in $\langle\tilde{\boldsymbol{\mu}}, \boldsymbol{\rho}^{\rm tr}\rangle$. In practice, however, one has only access to a finite-sample estimate $\hat{P}_N$, where N is the number of measurement shots per training state. Deviations of $\hat{P}_N$ from the true probability matrix lead to a *biased* estimator W for the input state, and thus to biased estimates for any target observable. This bias is the counterpart for QELMs of the systematic errors introduced by inaccuracies in the apparatus modelization in other estimation

approaches.

Effects of finite statistics on the test — Once the training has been completed, we have a fixed estimator to be used for appliations. Explicitly, this estimator is the function $b \mapsto (W_{i,b})_{i=1}^{N_{\rm obs}}$, which assigns the b-th column of W to each observation b. Or equivalently stated, if $N_{\rm test}$ measurement shots are taken in testing, with a fixed input state ρ, and the observed outcomes are $(b_j)_{j=1}^{N_{\rm test}}$, $b_j \in \{1, ..., N_{\rm out}\}$, then the resulting estimate for the i-th target observable is the empirical mean $\frac{1}{N_{\rm test}} \sum_{j=1}^{N_{\rm test}} W_{i,b_j}$. Denoting with $\hat{o}_{N_{\rm test}}$ this estimator, then by definition $\mathbb{E}[\hat{o}_{N_{\rm test}}] = {\rm Tr}(\rho \mathcal{O}_i)$, while its variance ${\rm Var}[\hat{o}_{N_{\rm test}}]$ is inversely proportional to $N_{\rm test}$. Standard statistical bounds like Chebyshev's or Hoeffding's can then be used to derive the statistics required to have a target estimation accuracy with high probability [5].

Overfitting — Overfitting is mitigated because QELMs rely on a linear model, which reflects the physical relationship between input density matrices and measurement probabilities. As long as one restricts the target functionals to linear ones (e.g., expectation values of observables) and uses linear regression for training, there is effectively no room for overfitting. If the training statistics are insufficient, however, the resulting estimator may carry a bias, leading to systematic estimation errors during testing — in just the same way as model inaccuracies lead to biases for traditional methods.

Structure of training states — To reconstruct *any* observable at the testing stage, the training states must span the space of all density matrices. Formally, the real linear span of $\{\rho_i^{\rm tr}\}_{i=1}^{N_{\rm tr}}$ must have dimension d^2, where d is the dimension of the relevant Hilbert space. Consequently, d^2 random pure states are in principle sufficient to train a QELM. However, as discussed in [47], using more than d^2 states can improve numerical stability in the corresponding linear regression. Nonetheless, one may successfully train a QELM with less than d^2 states if the same restricted subspace of states is also used for testing. The only requirement is that each testing density matrix can be in the linear span of the training ones. This enables more efficient training when only a limited set of states is of interest. Additional details on the training states employed in our experiments are provided in section S2 C.

S2. EXPERIMENTAL IMPLEMENTATION DETAILS

A. Departures from the basic model

In this section, we discuss some details of our experimental implementation that deviate from the standard QELM formalization outlined earlier. We examine these details for completeness, although they ultimately have no practical impact on the overall state estimation protocol.

Polarization projection — Our apparatus requires a polarization projection before OAM measurement because spatial light modulators only operate on photons with a fixed polarization, namely the one oriented as the tilting direction of the liquid crystal molecules in the active area of the device. As a result, the overall mapping from input density matrices to output probabilities is not strictly a quantum channel followed by a POVM. Instead, it can be modeled as a non-trace-increasing completely positive map, followed by a projective measurement. Equivalently, we can describe the "effective POVM" describing the apparatus as a strict subset of a full POVM. This does not affect our formal framework or training procedure but rather just increases the amount of data (statistics) required for both training and testing [52].

Post-selection measurements — Because we operate in a post-selection configuration, we do not have access to the total number of input states generated by the source. Instead, we record only the raw photon counts at the output, meaning that the number of copies of each input state is not directly measured, and rely instead on detected events — specifically, coincidence counts defined as the simultaneous triggering of two APDs within a given time window. Standard estimation methods, including state tomography, depend instead on accurately estimating output probabilities. Consequently, fluctuations or inaccuracies in the number of input states introduce significant estimation errors and make it impossible to distinguish between apparatus losses and variations in the source generation frequency. By contrast, QELMs do not require explicit knowledge of the total input count; as long as the overall count rate remains sufficiently stable, the QELM automatically learns the appropriate scaling factor to accurately extract the desired information from the measured counts. We further explore these aspects in section S4.

QELM training with counts vs frequencies — A potential issue arises if only experimental detection counts, rather

than normalized frequencies, are available during training. In our photonic implementation, the total number of photon pairs injected into the system is not directly accessible but can only be inferred from a precise calibration of the overall system efficiency. Although we perform coincidence detection to identify successful photon-pair events, in the absence of heralding and due to non-negligible losses and detectors inefficiencies, many generated pairs are lost before detection. Therefore, a more general description relies only on the number of measured events. The ideal estimator W, derived from normalized frequencies in the limit of infinite training statistics, is given by $W = \langle \mathcal{O}, \rho^{\rm tr}\rangle \langle \tilde{\mu}, \rho^{\rm tr}\rangle^+$. This operator implicitly depends on N through the term $\langle \tilde{\mu}, \rho^{\rm tr}\rangle$, which contains the output probabilities. However, if only the detection counts N_b corresponding to each outcome b are available, we must instead construct a "counts matrix", which for large N approximates $N\langle \tilde{\mu}, \rho^{\rm tr}\rangle$. Since the computation of W involves the pseudoinverse of this matrix, the learned estimator obtained from counts ends up being approximately scaled by a factor $1/N$. Consequently, if this estimator is applied to test states corresponding to a different total number of input states N', the resulting predictions are systematically scaled by a factor N'/N, introducing an unwanted bias. A practical way to mitigate this issue is to normalize the counts, replacing N_b with $N_b/\sum_{b'} N_{b'}$ during both training and testing. However, normalization can inject unwanted nonlinearities in the training when the experiment suffers from significant photon losses, which is common in realistic scenarios where $\sum_{b'} N_{b'} \ll N$. Still, under the reasonable assumption that photon losses occur independently of the input state, one can approximate $\sum_{b'} N_{b'} \approx \eta N$, where $\eta \ll 1$ represents the overall transmission efficiency of the apparatus. Since η is a fixed property of the setup and does not depend on measurement statistics, normalizing the counts does not introduce substantial additional error. Thus, this approach provides a reliable way to ensure consistency between training and testing, even when measurements are performed under different statistical conditions. In our experiments, training and test statistics are comparable, $N' \simeq N$, and we thus do not observe a substantial difference in the results obtained with and without normalizing experimental counts. However, this observation tells us that QELMs can be used even in cases where training and test datasets are measured with different statistics.

Implementation of projective OAM measurement — Another practical challenge in our experimental implementation is related to the single-setting nature of the measurement stage. Indeed, our setup cannot directly perform a projective measurement in the OAM basis $\{|m\rangle\}_{m=-2}^{2}$. Instead, we sequentially project onto each OAM state by using an SLM to transform that state into the fundamental TEM_{00} mode, which is then coupled into a single-mode fiber and detected with an APD. Although formally the resulting statistics differs from that given by a direct projective measurement, we find that in practice this difference is negligible. We can track this down to the fact that with sufficiently high statistics, a multinomial distribution is well approximated by a set of Poisson-distributed detection events, rendering the sequential measurement effectively equivalent to a single projective measurement.

B. Photon source

Photon-pair generation — The experimental setup used for generating input states is illustrated in Fig. 2-(a) of the Main Text. Photon pairs at 808 nm are produced via spontaneous parametric down-conversion (SPDC) in a periodically poled potassium titanyl phosphate (ppKTP) nonlinear crystal, pumped by a continuous-wave laser at 404 nm and placed within a Sagnac interferometer. The poling of the crystal is engineered to have a collinear emission with a type II phase-matching condition, enabling the process $|H\rangle_p \to |H\rangle_i |V\rangle_s$ where the subscripts denote the pump, idler, and signal photons. By adjusting the pump polarization with a half-wave plate (HWP), various two-photon states can be generated, including both separable and entangled states. We will refer to the states generated by the source as *reference states*, as the "input states" that are fed into the model are obtained by applying local unitary operations to these reference states. Changing the reference states thus allows to switch between generating separable or entangled states. Separable states are obtained by pumping the crystal with a horizontally polarized beam, which generates photons in only one arm of the interferometer. As reference separable states we used $|\Psi_R^{\rm sep,1}\rangle \equiv |H, V\rangle$ and $|\Psi_R^{\rm sep,2}\rangle \equiv |V, V\rangle$, where the latter is obtain from the first by using an additional HWP before entering the QW setup. Conversely, maximally entangled states are produced by using a diagonally polarized pump beam, resulting in a superposition of clockwise and counterclockwise emissions. The maximally entangled states generated by our source have the form $|\Psi_R^+\rangle = \frac{1}{\sqrt{2}}(|H\rangle_1|V\rangle_2 + |V\rangle_1|H\rangle_2)$, where the subscripts indicate the output ports of the interferometers. We furthermore investigated the generation and the model performance when the pump polarization is set in such a way as to generate *partially* entangled reference states. In these cases, we used the states $|\Psi_R^+\rangle_{p_1} = 1/\sqrt{4}\,|H\rangle_1\,|V\rangle_2 + \sqrt{3/4}\,|V\rangle_1\,|H\rangle_2$ and $|\Psi_R^+\rangle_{p_2} = 1/\sqrt{5}\,|H\rangle_1\,|V\rangle_2 + \sqrt{4/5}\,|V\rangle_1\,|H\rangle_2$. The results of this analysis are reported in section S3.

Quality assessment of generated states — To assess the quality of the states generated by the source we performed

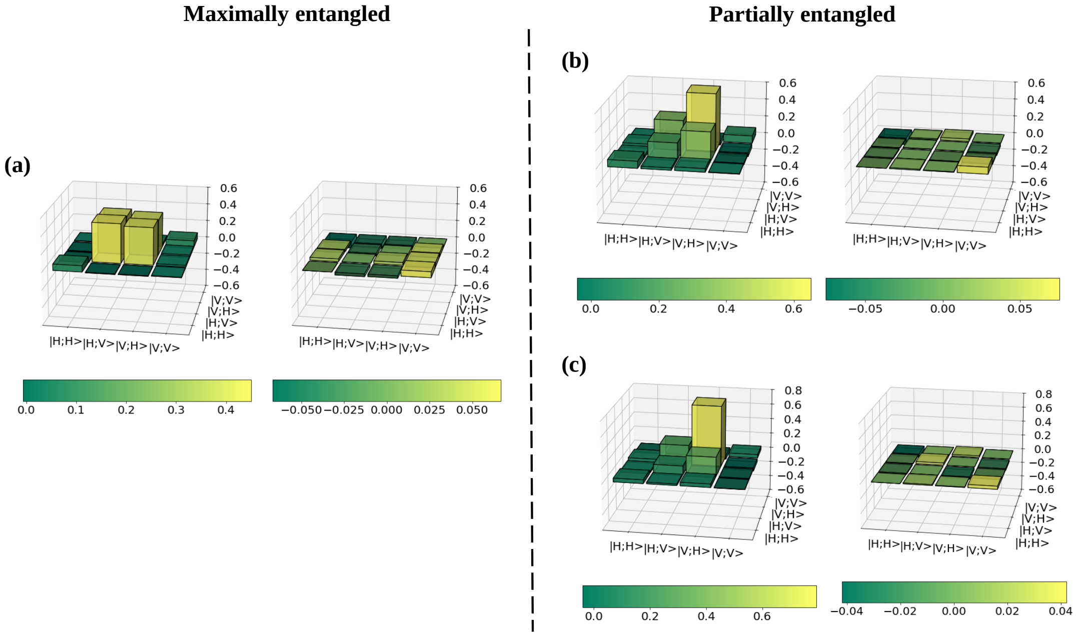

Figure S1. **Input states density matrix.** Experimentally reconstructed real (left) and imaginary (right) parts of the density matrix associated with the states generated by the SPDC source: (a) entangled $\left|\Psi_R^+\right\rangle$, (b) partially entangled $\left|\Psi_R^+\right\rangle_{p_1}$ and (c) partially entangled $\left|\Psi_R^+\right\rangle_{p_2}$.

State	$\mathcal{F}$	$\mathcal{S}$
$\left\|\Psi_R^+\right\rangle$	0.923 ± 0.002	2.685 ± 0.013
$\left\|\Psi_R^+\right\rangle_{p_1}$	0.905 ± 0.002	2.432 ± 0.013
$\left\|\Psi_R^+\right\rangle_{p_2}$	0.927 ± 0.001	2.301 ± 0.012

Table S1. **Quality of reference states.** We report here the fidelity $\mathcal{F}$, obtained from the experimentally reconstructed density matrix, and the Bell parameters $\mathcal{S}$ of the CHSH inequality violation for all the states under analysis. The errors are computed by assuming a Poissonian statistics on the experimentally measured counts.

full quantum state tomography on them. In particular, we measured the expectation values of the combination of the three Pauli operators $\{\sigma_x, \sigma_y, \sigma_z\}$ and the identity $\mathbb{1}$ on the two-photon states, and used a maximum-likelihood approach to retrieve their density matrix. The real and imaginary parts of the reconstructed density matrices are shown in fig. S1. Moreover, we also verified the violation of the CHSH inequality to asses the entangled nature of the produced photon pairs. The values obtained for the fidelities and the Bell parameter S are collected in table S1 for all the states under analysis.

Estimated experimental statistics — The photon-pair source is pumped with a continuous-wave laser having a power $P \approx 8\,\mathrm{mW}$, and yielding a coincidence rate of $\mathrm{cc_s} \approx 20\,\mathrm{kHz}$ at the output of the Sagnac interferometer. Using a coherent 808 nm laser we measure the transmission efficiencies of all the elements present in the experimental setup. In particular, for the q-plates and the whole QW we obtained efficiencies $\eta_{\mathrm{QP}} \approx 0.80$ and $\eta_{\mathrm{QW}} \approx 0.56$, respectively. At the QW output, we use a HWP, a QWP, and a PBS to project the polarization degree of freedom, which introduces an average signal loss of $\eta_{\mathrm{proj}} \approx 0.5$ per photon. We then perform projective measurements in the OAM space by using a combination of an SLM and single-mode fibers coupling. For these we measured average efficiencies of $\eta_{\mathrm{SLM}} \approx 0.78$ and $\eta_{\mathrm{SMF}} \approx 0.4$, respectively. Combining all these factors, the estimated coincidence rate for each of the 25 output events is

$$\mathrm{cc_{teo}} = \eta_{\mathrm{QW}}^2 \eta_{\mathrm{proj}}^2 \eta_{\mathrm{SLM}}^2 \eta_{\mathrm{SMF}}^2 \mathrm{cc}_s \approx 6.1\,\mathrm{Hz}. \tag{S1}$$

By comparison, for the **E1** experimental configuration we observed a rate of $\approx 2.9\,\mathrm{Hz}$. In this experiment, we set the acquisition time to 8 seconds per projection, then, repeating each measurement 2 times to increase the statistical significance. The overall average counts per state is then found to be 1117 ± 76 cc/state, summing the observed counts over all 25 outcomes.

C. Experimental configurations

We report here a summary of the experimental configurations used to acquire the reported data. We considered several scenarios to test how performances are affected by different model parameters, choice of training states, and collected statistics.

Experimental settings — We present data relative to 6 distinct experimental datasets, labeled **E1**, **E2**, **E3**, **E4**, **E5**, **E6**. These correspond to different reservoir and training configurations. We used three reservoir configurations, labeled R1, R2, and R3. R1 is the reservoir obtained setting the quantum walk waveplates angles in order to minimize the trace of the inverse frame superoperator corresponding to the effective POVM. This is a procedure previously outlined in [52] that provides the reservoir configuration corresponding to the minimal MSE averaged over all possible target observables. R2 is a reservoir configuration obtained from R1 by swapping the angles of the QWPs implementing the coin operator in the second step of the QW evolution, and R3 is yet another reservoir configuration obtained setting the waveplates angles totally at random. A breakdown of the characteristics of each dataset presented is provided in table S2. The exact values used for the reservoir configurations, as well as the experimental data used to train and test the QELM in each configuration, and the code used to generate the reported data, is available at `https://github.com/salvatore-lorenzo/arXiv-2502.18361-QELM_WITNESS`.

Input states — The input states used to train the model were generated by applying local unitary operations — realized with a HWP-QWP pair on each QW — to the reference states. Each such single-qubit unitary has thus the form $U(\phi,\theta) = \mathrm{QWP}(\phi)\mathrm{HWP}(\theta)$, for some pair of angles $\phi,\theta \in \mathbb{R}$. Tuning the angles in each of these four waveplates we can generate a large variety of possible input states. In all cases, we generated the input states sampling uniformly random angles for each of the waveplates. For our results, we either used the same angles on both sides, or we sampled the random angles independently on the two arms. In the first case, the input states thus have the form $(U(\phi,\theta) \otimes U(\phi,\theta)) \left|\Psi_R\right\rangle$ with $\left|\Psi_R\right\rangle$ the reference state. This is the scenario considered in the datasets **E1**, **E2**, **E3**, **E5**, **E6**. On the other hand, in **E4** we analyzed the performances in the case where input states take the form $(U(\phi_1,\theta_1) \otimes U(\phi_2,\theta_2)) \left|\Psi_R\right\rangle$, with independently sampled $\phi_1,\theta_1,\phi_2,\theta_2$.

Singular values and model trainability — A practical way to assess the structure of a set of states from the perspective of model trainability is to examine the dimension of the space it spans. This can be done by inspecting the singular values of the matrices whose rows are the vectorized density matrices. Focusing on the cases where training and test states are comprised entirely of separable and entangled states, respectively, we thus define the matrices M^{sep}, M^{ent}, and M^{all} to contain separable, entangled, and all states, respectively. More precisely, M^{sep} is the matrix whose k-th row is the vectorization of the k-th separable state. The number of nonzero singular values of M^{sep}, which also equals $\mathrm{rank}(M^{\mathrm{sep}})$, is then the dimension of the real linear span of $\{\rho_k^{\mathrm{tr}}\}_{k=1}^{N_{\mathrm{tr}}}$, when the training is comprised entirely of separable states. We similarly define M^{ent} and M^{all} for the entangled states alone and for all states, respectively. This yields the three numbers $\mathrm{rank}(M^{\mathrm{sep}})$, $\mathrm{rank}(M^{\mathrm{ent}})$, and $\mathrm{rank}(M^{\mathrm{all}})$, reported in table S2 for our various experimental configurations. These ranks reveal whether successful QELM training can be expected from a fundamental informational standpoint. In particular, $\mathrm{rank}(M^{\mathrm{sep}}) = 16$ means that the training separable states span the entire 16-dimensional space of density matrices in our setup, enabling the QELM to learn any feature of any state. Conversely, if $\mathrm{rank}(M^{\mathrm{sep}}) < \mathrm{rank}(M^{\mathrm{all}})$, which happens for **E2**, **E3**, **E5**, **E6**, certain features of the test states lie outside the linear span of separable states, requiring to add some entangled states in the training. Ideally, $\mathrm{rank}(M^{\mathrm{sep}}) = \mathrm{rank}(M^{\mathrm{all}})$, as is true for the **E1** and **E4**. On the other hand, higher $\mathrm{rank}(M^{\mathrm{sep}})$ corresponds to a higher resource requirement in terms of training statistics used per state. The best configuration choice thus ultimately depends on balancing these resource demands against the actual application one has in mind for the device.

Polarization projection — The fixed polarization state $\left|\eta\right\rangle = \cos(\theta_p)\left|H\right\rangle + e^{i\phi_p}\sin(\theta_p)\left|V\right\rangle$, on which the QW output states are projected before measuring their OAM distribution, is given by the same minimization on the frame superoperator reported above [52]. In particular, using a HWP, a QWP and a PBS to perform the polarization projection, we set the waveplates angles $\{\theta_{\mathrm{proj}}, \phi_{\mathrm{proj}}\}$ by solving the relation:

$$\mathrm{QWP}(\theta_{\mathrm{proj}})\,\mathrm{HWP}(\phi_{\mathrm{proj}})\left|\eta\right\rangle = \left|H\right\rangle \tag{S2}$$

Therefore, using the PBS to allow only the transmission of H-polarized photons, we actually perform a polarization projection on the wanted $\left|\eta\right\rangle$ state.

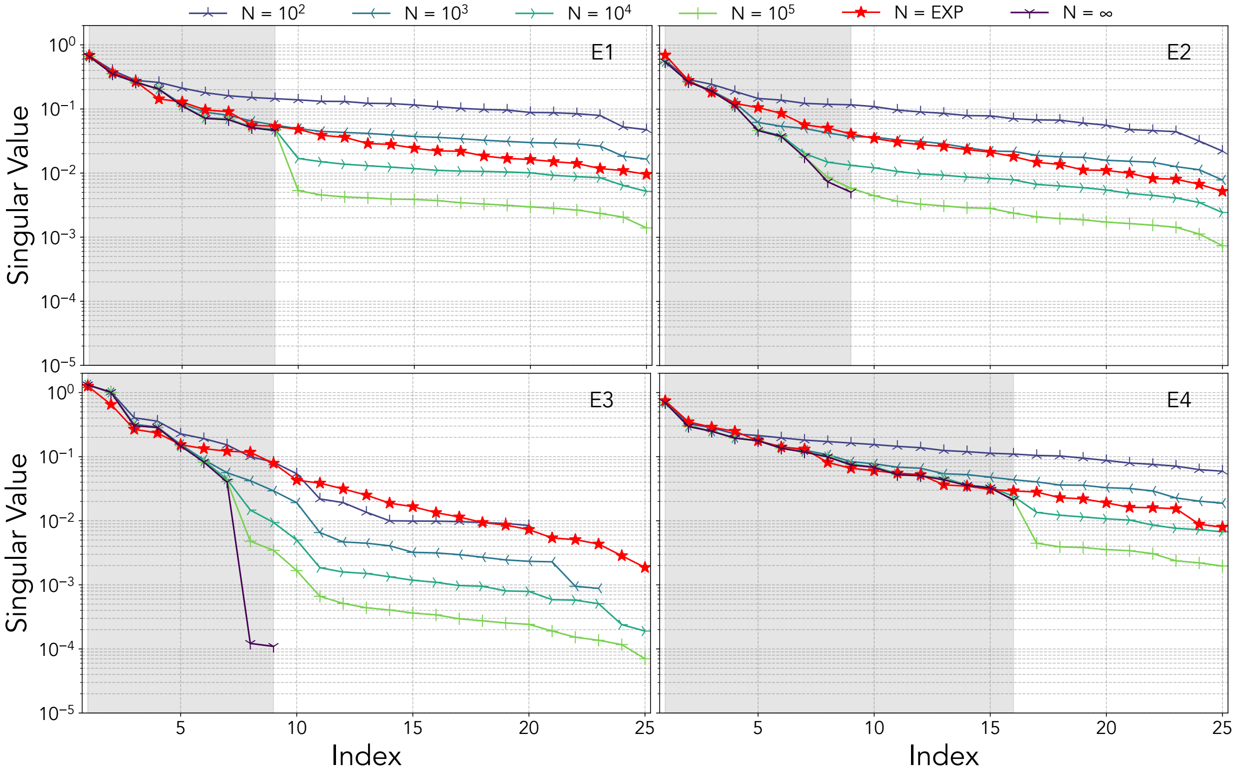

Figure S2. **Theoretical vs experimental singular values.** Singular values of the probability matrix $\langle \tilde{\boldsymbol{\mu}}, \boldsymbol{\rho} \rangle$ computed from the separable states in each of the four configurations **E1**, **E2**, **E3**, **E4**. For each configuration, we plot the singular values of the matrix obtained by sampling from $\langle \tilde{\boldsymbol{\mu}}, \boldsymbol{\rho} \rangle$ with statistics N, along with the $N = \infty$ (which corresponds to exact probabilities), and the singular values of the corresponding experimental counts matrix. In the experimental case, the matrix is normalized by the estimated number of states before the polarization projection, calculated as $N = 4\overline{N}$, where $\overline{N}$ is the averaged observed counts over all separable states. The shaded region highlights the number of nonzero singular values for the exact probability matrix, and thus corresponds to $\mathrm{rank}(M^{\mathrm{sep}})$ as reported in table S2. As N increases, the sampled singular values approach those of the $N = \infty$ curve. Configurations **E1** and **E4** use the R1 reservoir (which is optimized for information recovery), and correspondingly exhibit higher singular values, whereas **E2** and **E3** use less optimal reservoirs, and thus correspondingly smaller singular values. In **E3**, for instance, even $N = 10^5$ is insufficient to reproduce the smallest two singular values, implying the existence of two observables which to be recovered require an even larger amount of training statistics. The singular values from the experimental counts matrix roughly align with theoretical curves at $N \sim 10^3$, matching the typical count rates observed in the experiment.

S3. ADDITIONAL EXPERIMENTAL RESULTS

Singular values of counts matrices — A useful way to assess how readily information can be extracted from experimental data, given a choice of training states and reservoir, is to examine the spectrum of singular values of the matrix $\langle \tilde{\boldsymbol{\mu}}, \rho^{\mathrm{tr}} \rangle$, and its sampled counterpart obtained for different training statistics N. This analysis is presented in fig. S2, where we focus on the case of exclusively separable training states. When using exact probabilities and assuming an informationally-complete effective POVM, the number of nonzero singular values equals $\mathrm{rank}(M^{\mathrm{tr}})$. Although this rank reveals the theoretical maximum amount of information contained in the measurement data, the magnitudes of the singular values themselves are equally important. In particular, small singular values correspond to observables that require large training statistics for accurate reconstruction. This viewpoint aligns with [47], where the condition number of the same matrix is used to quantify the ill-conditioning of the QELM training problem. Note that $\langle \tilde{\boldsymbol{\mu}}, \rho^{\mathrm{tr}} \rangle$ can have at most 16 nonzero singular values, corresponding to the dimension of the space of input density matrices. However, when sampling with finite N, we get more than 16 nonzero singular values due to sampling noise. Consequently, any singular values of similar order to those beyond the 16th effectively correspond to observables that cannot be accurately estimated from the available measurement data, since the associated data is indistinguishable from the statistical noise.

Additional data for **E1** — We report in figs. S3 and S4 the estimation performances using Pauli matrices as target observables, as well as for the four entanglement witnesses built with the Bell states, in **E1**. In particular, fig. S3 provides a more complete picture of how our apparatus maps input information into the measured degrees of

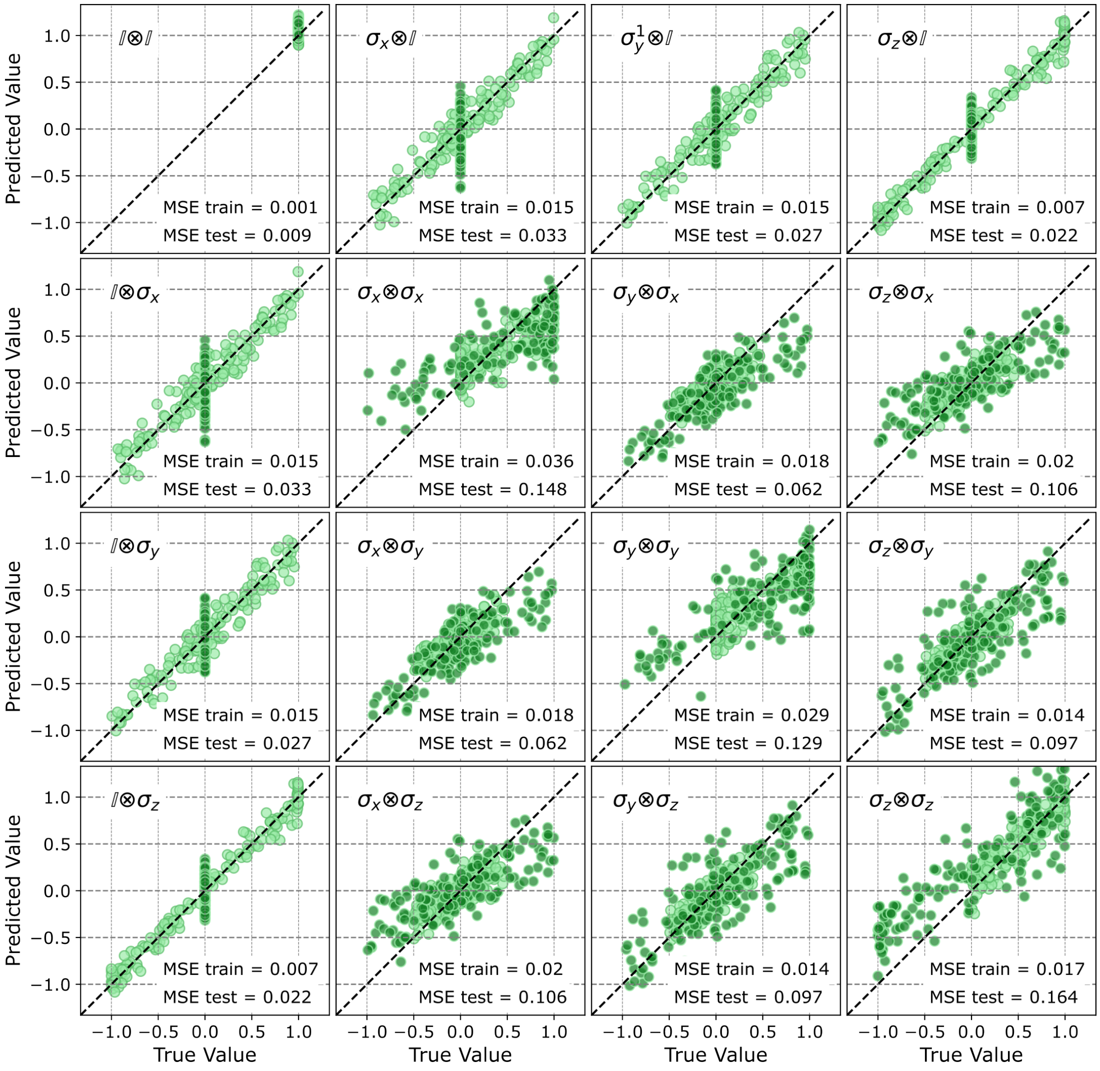

Figure S3. **Reconstruction MSEs of Pauli observables.** Estimation performance in **E1** for target observables that are products of local Pauli matrices, i.e., $\sigma_j \otimes \sigma_k$ with $j, k \in \{0, 1, 2, 3\}$ and $\sigma_0 \equiv I$. In all cases, the training set consists entirely of separable states, while the test set consists entirely of maximally entangled states. Each plot displays both the training predictions (light-green dots) and the test predictions (dark-green dots). For local Pauli observables of the form $\sigma_i \otimes I$ or $I \otimes \sigma_i$, $i \in \{1, 2, 3\}$, the true expectation values are zero, resulting in the observed vertical clustering of predicted values for the maximally entangled states. Within each plot, we report the average MSEs for training states and for test states. These results clearly show that the estimation MSE depends on the choice of target observable, in agreement with theoretical expectations. In the limit of very large training and test sample sizes, we expect all points in these scatter plots to align along the main diagonal.

freedom. We observe that local observables are predictably easier to reconstruct when using separable states. The vertical lines corresponding to the test entangled state are due to testing states being maximally entangled, meaning they always have zero expectation value on local Pauli observables.

Training with asymmetric input states — We next investigated estimation performances in **E4** (cf. table S2). In this case we used a input state preparation scheme where the waveplate angles used to prepare the input states of the two QWs are chosen independently from each other. This naturally increases both the complexity of the estimation task and the required experimental statistics. We once again evaluate the estimation accuracy for various entanglement witnesses and Pauli observables. In fig. S5-(a) we show that $\mathcal{W}_{\Phi^+}$ is still estimated effectively in this scenario, though the performance is somewhat lower than in **E1**. This reduction arises from the higher training-statistics requirement imposed by a larger training set, rather than from any fundamental limitation of the method. Figure S5-(b) reports the exact expectation values $\langle \mathcal{W}_{\Phi^+} \rangle$ for random states, where the waveplate angles used to prepare the input states entering the two QWs differ by an amount drawn from a normal distribution $\mathcal{N}(0, \delta^2)$. Under these conditions, fewer of the generated states exhibit negative $\langle \mathcal{W}_{\Phi^+} \rangle$. Overall, fig. S5 illustrates how the accuracy of entanglement witnessing declines when states are generated at varying angles and the available statistics are limited. Nevertheless,

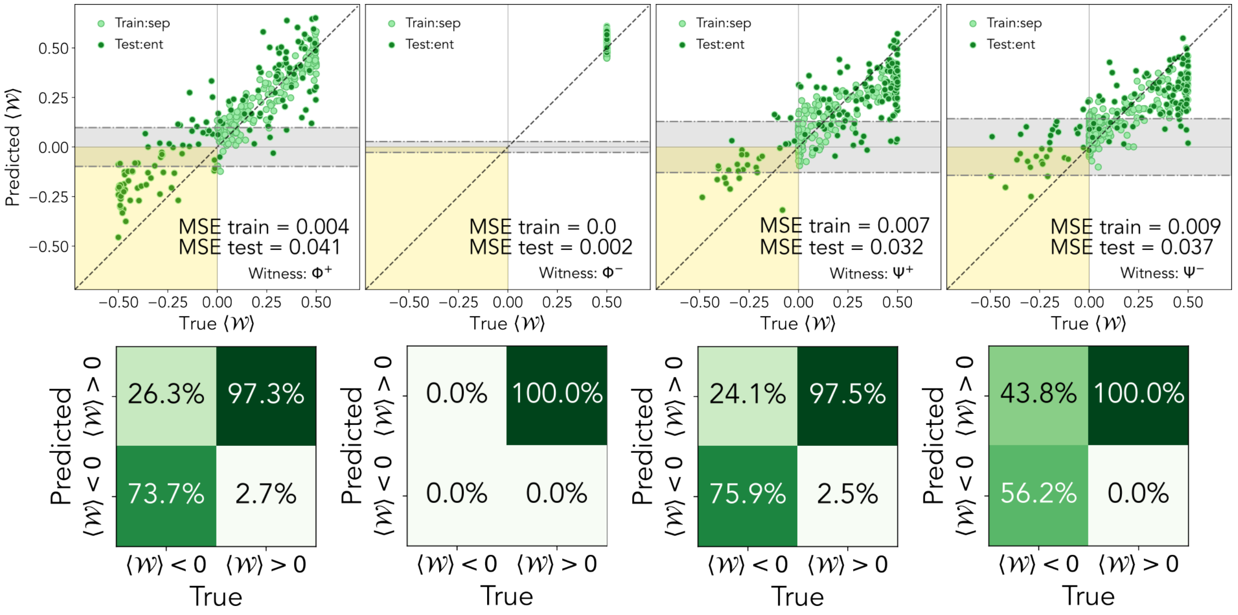

Figure S4. **Reconstruction MSEs for entanglement witnesses.** Estimation performance in **E1** for the four Bell witnesses $\mathcal{W}_i$. As before, the training contains only separable states, while the test contains only maximally entangled states. Each plot shows both the training predictions (light-green dots) and the test predictions (dark-green dots). The results for the Φ^+ witness are the ones also reported in the main text. The trivial results for the Φ^- witness are due to the particular symmetry of states generated in the **E1** configuration, which all give the same value for $\langle \mathcal{W}_{\Phi^-} \rangle$. Within each plot, we report the mean squared error (MSE) averaged over all training states and all test states. Furthermore, the shaded gray area has width corresponding to the average MSE estimated from the training dataset. Points with predicted $\langle \mathcal{W} \rangle$ below this region can be more reliably considered as certifiably entangled.

Label	Reservoir type	Reference entangled states	Reference separable states	Generation angles	States ranks
**E1**	R1	$\lvert\psi_R^+\rangle$	$\lvert VV\rangle$	Equal	9-9-9
**E2**	R2	$\lvert\psi_R^+\rangle,\ \lvert\psi_R^+\rangle_{p1}$	$\lvert VH\rangle$	Equal	9-6-10
**E3**	R3	$\lvert\psi_R^+\rangle$	$\lvert VH\rangle$	Equal	9-6-10
**E4**	R1	$\lvert\psi_R^+\rangle$	$\lvert VH\rangle$	Different	16-10-16
**E5**	R2	$\lvert\psi_R^+\rangle$	$\lvert VH\rangle$	Equal	9-6-10
**E6**	R2	$\lvert\psi_R^+\rangle,\ \lvert\psi_R^+\rangle_{p2}$	$\lvert VH\rangle$	Equal	9-6-10

Table S2. **Summary of experimental configurations** For each configuration Ei we report which of the three reservoir configurations was used; the reference entangled and separable states; whether the preparation angles used to generate input states were equal or different for the two QWs; under "states ranks", the ranks of M^{sep}, M^{ent}, and M^{all}, corresponding to the dimension of the span of separable and entangled states, and of all states.

the same performance attained in **E1** can be recovered with a larger training-data budget, demonstrating that the drop in accuracy is not intrinsic but rather a consequence of increased training demands.

Training with different reference separable states — We also investigated how the estimation performance changes when a different reference separable state is used prior to the state preparation stage. A characteristic of the QW dynamics we employ is that, when using the reference state $\lvert VH\rangle$, as done in **E2**-**E6**, only a strict subspace of states is generated when the same angles are used on both preparation branches, due to the specific symmetries of the dynamics. In this scenario, the input states have the form $(U \otimes U)\lvert VH\rangle$ and $(U \otimes U)\lvert\Psi_R^+\rangle$ for separable and entangled states, respectively, where U is a random single-qubit unitary. This can be verified by studying the ranks of M^{sep} and M^{all}, which are also reported in table S2. When the reference separable state is $\lvert VH\rangle$, we have $\text{rank}(M^{\text{all}}) > \text{rank}(M^{\text{sep}})$, indicating that some test entangled states span directions the QELM never had a chance to learn about during training. This effect is showcased in fig. S6, where we observe that even in the theoretical simulation there is a shift between the true and predicted expectation values of the target witness $\mathcal{W}_{\Phi^+}$. This constant bias arises because all entangled states in the test share a fixed expectation value on the single observable present in the test but missing in the training dataset. In fact, adding even just one single entangled state to the training dataset is sufficient to characterize that missing dimension, and thus fix the bias. On the other hand, this issue does not arise when the reference separable state is $\lvert VV\rangle$, owing to the different symmetry properties of the reservoir in this case. These

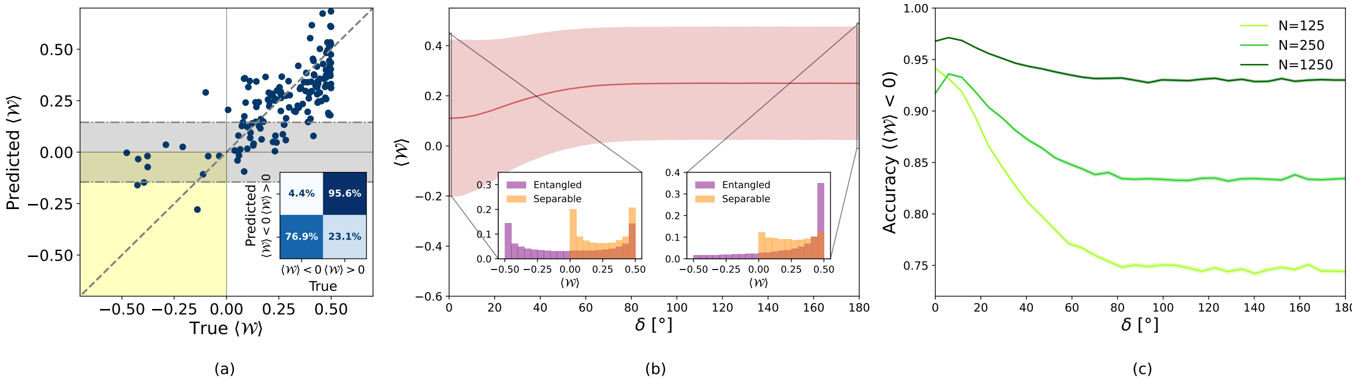

Figure S5. **Effect of waveplates angles mismatch.** Estimation performances to reconstruct $\mathcal{W}_{\Phi^+}$ as we move from the same angle condition, by adding a mismatch extracted from a normal distribution $\mathcal{N}(0, \delta^2)$ in the input stage HWP and QWP of one of the two quantum walks (see Fig. 2 of the main text). (a) Estimation performance in **E4**. We collected 300 quantum states, dividing them into training and test sets of 150 elements. The plot shows the retrieved witness values and the confusion matrix over the test set. This dataset achieves values of the MSE equal to 0.021 and 0.022, respectively for the training and test set, where the square root of the former is also reported as a gray shaded area in the plot. (b) The plot shows the theoretical witness expected values for the states in the dataset as a function of the angle mismatch δ, where the red solid line and shaded area respectively represent their mean and standard deviation. The purple (orange) histogram corresponds to the witness estimations over the entangled (separable) states, and it showcases how the overall distribution moves towards positive values when δ increases, reducing in this way the negative instances in the dataset. (c) Theoretically simulated estimation performance in retrieving negative values over the test set. The plot reports the mean accuracy over 5000 angles mismatch instances (randomly selected from $\mathcal{N}(0, \delta^2)$), for three different statistics conditions (125, 250, 1250 signals). The results highlight that the estimation accuracy tends to decrease when δ increases, with a contribution that is less relevant for higher statistics. The error band associated with each of the three curves is calculated as the standard deviation of the mean, which is too small to be evident in the plot.

findings represent further evidence that the way training states are generated can be crucial from an estimation perspective, particularly when the underlying reservoir dynamics present specific symmetries and do not uniformly spread information at random into the output degrees of freedom.

Training with partially entangled reference states — As discussed in section S2 B, the Sagnac source allows to produce photon pairs with varying degrees of entanglement by adjusting the pump polarization. We leveraged this to also investigate, in **E2** and **E6**, the estimation performance when using partially entangled states, focusing in particular on the states $|\Psi^+\rangle_{p_1}$ and $|\Psi^+\rangle_{p_2}$ described above. For each of these reference states, we collected a dataset of 249 states in the first case and 357 in the second, both equally divided among maximally entangled, partially entangled, and separable states. Both these datasets are generated considering the R2 reservoir configuration and using identical angles at the state preparation stage. We denote these two datasets as p_1 and p_2, respectively, indicating which partially entangled state they contain. To test the model's resilience to undetected experimental defects in entangled-photon generation, we emulate an imperfect source that randomly produces either maximally or partially entangled photon pairs, without our knowledge. We use a training dataset comprising 50% of the total dataset and intentionally mislabel the partially entangled states as maximally entangled. The predicted expectation values of $\mathcal{W}_{\Phi^+}$ are shown in fig. S7-(a,b) for the p_1 and p_2 datasets, respectively. Moreover, we examine how varying degrees of entanglement in the states $|\Psi^+\rangle$, $|\Psi^+\rangle_{p_1}$, and $|\Psi^+\rangle_{p_2}$, affect the model performance. As reported in fig. S7-(c), the accuracy remains high across all three classes of entangled states, even for those with $\langle \mathcal{W}_{\Phi^+} \rangle < 0$. These results indicate that robust estimation performance can still be maintained even when undetected imperfections reduce the amount of entanglement.

Additional data — Additional experimental results, including the MSEs for all Pauli observables and Bell witnesses for all the reported experimental configurations, as well as the code used to generate all reported data, is available in the GitHub repository `https://github.com/salvatore-lorenzo/arXiv-2502.18361-QELM_WITNESS`.

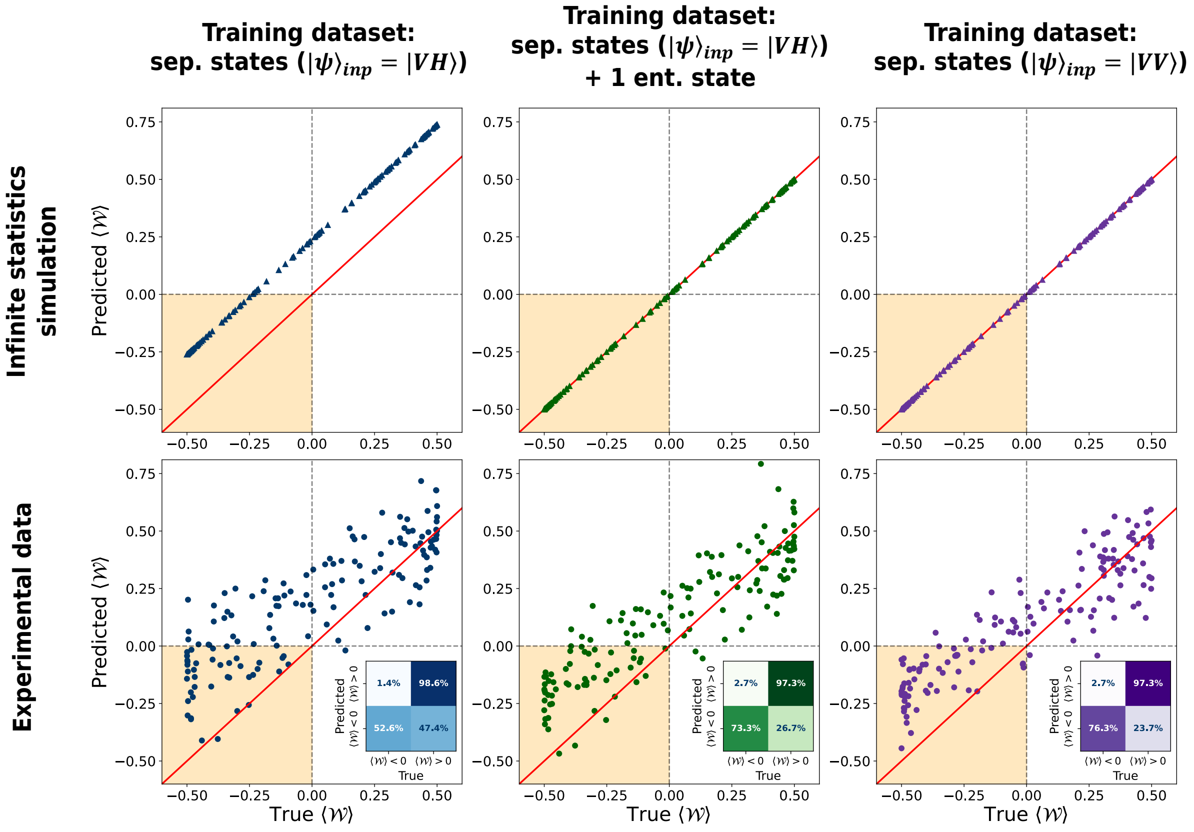

Figure S6. **Predictions with different separable input states.** Estimation performances to reconstruct $\mathcal{W}_{\Phi^+}$ training with separable states and testing with entangled states. The upper row reports the simulated case with infinite training statistics, while the lower displays the experimental one. The experimental data in the first two plots comes from **E5**, while the third from **E1**. The first column reveals how, also in the infinite statistic case scenario, using $|HV\rangle$ as input separable state doesn't enable us to retrieve the correct values of the witness, resulting in a constant shift in the estimation. This can be solved by adding just one entangled state to the training set (middle column), as evident also from the accuracy performance in the experimental regime. The third column shows that using $|VV\rangle$ as separable reference state allows to correctly estimate $\mathcal{W}_{\Phi^+}$.

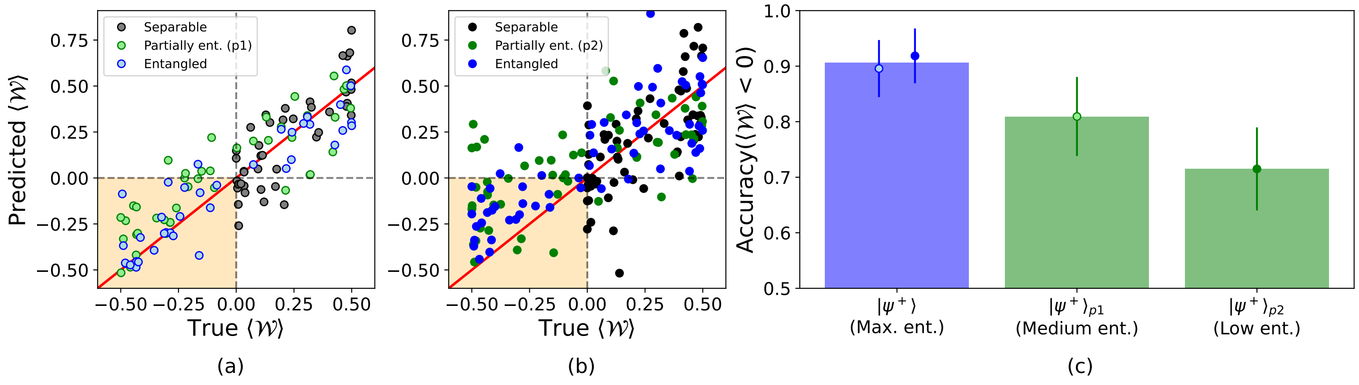

Figure S7. **Estimation performance with partially entangled reference states.** Panels **(a)** and **(b)** show the predicted expectation values of $\mathcal{W}_{\Phi^+}$ when using as reference entangled states the partially entangled $|\Psi^+\rangle_{p_1}$ and $|\Psi^+\rangle_{p_2}$, as defined in section S2 B. More specifically, we intentionally mislabel the partially entangled states during training, to assess the model's robustness to mislabeling errors. Panel **(c)** reports the accuracy in detecting states with $\langle \mathcal{W}_{\Phi^+} \rangle < 0$ for the three entanglement cases. The bar heights show the average accuracy over 10^4 random splits of the training and test sets, with the error bars representing the corresponding standard deviations. Green bars correspond to partially entangled states, while the blue bar represents maximally entangled states. Within each color, empty dots refer to the p_1 dataset and filled dots to the p_2 dataset. The heights of the green bars reflect the average accuracy specific to $|\Psi^+\rangle_{p_1}$ and $|\Psi^+\rangle_{p_2}$, whereas the blue bar's height is the mean accuracy across both datasets for the maximally entangled states.

S4. BENCHMARKS WITH ALTERNATIVE APPROACH

Description of shadow tomography — To benchmark the estimation performances of our QELM-based approach it is useful to compare them with what we would have obtained with alternative methods relying on prior knowledge of the experimental apparatus. In particular, shadow tomography [5,8,9,12] is a general methodology for computing estimators of target observables, in any measurement scenario that can be described by a POVM applied to output states after some evolution. More specifically, shadow tomography provides the optimal unbiased estimator — meaning that it produces the unbiased estimator with the lowest possible average variance. Shadow tomography thus serves as a natural benchmark for QELM-based estimation, since both approaches aim to enable efficient estimation of specific properties of input states without requiring a full tomographic reconstruction.

How does shadow tomography work — The core difference between shadow tomography and QELM-based reconstruction lies in the way the estimator is constructed. In QELMs, we use a training dataset of states and the corresponding measurement outcomes. In contrast, shadow tomography — like state-tomography techniques — requires no training dataset but instead demands explicit and accurate knowledge of the entire measurement apparatus. Formally, we can compute the shadow tomography estimator associated with any POVM $\tilde{\boldsymbol{\mu}}$. In our case, $\tilde{\boldsymbol{\mu}}$ represents the effective measurement describing everything that happens to the input states, up to the final measurement. Given this POVM, we define the *frame superoperator* $\mathcal{F}$ as $\mathcal{F}(X) \equiv \sum_b \mathrm{Tr}(\tilde{\mu}_b X)\,\tilde{\mu}_b$, for any operator X. We then compute the *dual measurement frame* elements $\tilde{\mu}_b^\star \equiv \mathcal{F}^{-1}(\tilde{\mu}_b)$, where $\mathcal{F}^{-1}$ is the inverse of $\mathcal{F}$, understood as a linear operator acting on the space of Hermitian operators. As shown in [9], $\tilde{\mu}_b^\star$ can be interpreted as the optimal unbiased estimator for the state. Given any target observable $\mathcal{O}$, the corresponding optimal unbiased estimator is then defined by $\hat{o}(b) \equiv \mathrm{Tr}(\mathcal{O}\,\tilde{\mu}_b^\star)$.

How to apply shadow tomography in our case — Concretely, estimating target observables via shadow tomography in our experiment would thus entail computing analytically the effective POVM $\tilde{\boldsymbol{\mu}}$, and use it to build $\tilde{\mu}_b^\star$ with the recipe outlined above. The reservoir dynamic we employ, as previously discussed in section S2 C, has the form $(I_{\mathrm{OAM}} \otimes \langle\eta|)V\,|\Psi\rangle$, with $|\eta\rangle$ the polarization state on which we project at the end, $|\Psi\rangle$ the input bipartite polarization state, and $V \equiv V_1 \otimes V_2$ the isometry corresponding to the two QWs. The corresponding effective POVM is given by $\tilde{\mu}_b = V^\dagger(|b\rangle\langle b| \otimes |\eta\rangle\langle\eta|)V$, where $|b\rangle$ is the OAM computational basis, and this is then used to compute $\tilde{\mu}_b^\star$.

Knowing counts vs frequencies — We previously discussed in section S2 A why and how QELMs can operate without explicit knowledge of the total input statistics N, relying solely on raw detection counts. This issue is exacerbated for alternative methods such as shadow tomography or standard tomography, where there is no training dataset to help estimate N. Consequently, one must in these cases rely on an experimental estimate of the overall transmission rate, which might bring up additional estimation errors. To avoid an unfair comparison and artificially pessimistic outcomes for shadow tomography, we instead report the MSE as a function of the guessed N and then take its minimum value. It is to be noted, however, that this minimum depends on the target observable, implying different values of N for different observables — an obviously unphysical scenario. Hence, the values obtained should be viewed as lower bounds on what would be achieved if the true N were known. In other words, they represent a best-case scenario — that is, a lower bound — for the performance of linear-estimator-based methods that rely on measurement probabilities at the output.

Performances of theoretical model of the apparatus — We report in fig. S8 the MSEs as a function of the possible values of the unknown input statistics N, for the experimental configurations **E1**, **E2**, **E3**, **E4**, for the four Bell witnesses, and when applied to the subsets of separable and entangled states, separately. Explicitly, the MSE is calculated for each target observable $\mathcal{W}$ and statistics N, averaging over the states as

$$\mathrm{MSE}(\mathcal{W};N) = \frac{1}{N_{\mathrm{test}}}\sum_{i=1}^{N_{\mathrm{test}}}\left|\sum_{b=1}^{N_{\mathrm{out}}}\hat{o}(b)\frac{N_b}{N} - \mathrm{Tr}(\mathcal{W}\rho_i^{\mathrm{test}})\right|^2. \tag{S3}$$

Here, N_{test} is the number of test states ρ_i^{test} used to assess the accuracy of the shadow tomography estimators (note that with this method, there is no training dataset to use). The estimator $\sum_b \hat{o}(b)\frac{N_b}{N}$ returns the best estimate for the expectation value of $\mathcal{W}$, when N_b counts are observed for each outcome b, and the overall statistics is N. We use the experimental counts as values of N_b for each training state ρ_i^{test}, and try different values for the unknown value of N. For large N, this produces the trivial estimator $b \mapsto 0$, and thus in this limit $\mathrm{MSE}(\mathcal{W};N) \to \frac{1}{N_{\mathrm{tr}}}\sum_{i=1}^{N_{\mathrm{tr}}}\mathrm{Tr}(\mathcal{W}\,\rho_i^{\mathrm{test}})^2$, which explains the saturation observed in the MSE curves. Alongside $\mathrm{MSE}(\mathcal{W};N)$ for varying N, we also report $\mathrm{MSE}(\mathcal{W}) \equiv \min_N \mathrm{MSE}(\mathcal{W};N)$, which serves as a lower bound on the MSE achievable via shadow tomography. Note that this minimum depends on the target observable, which means that it cannot be used as a reliable estimate for

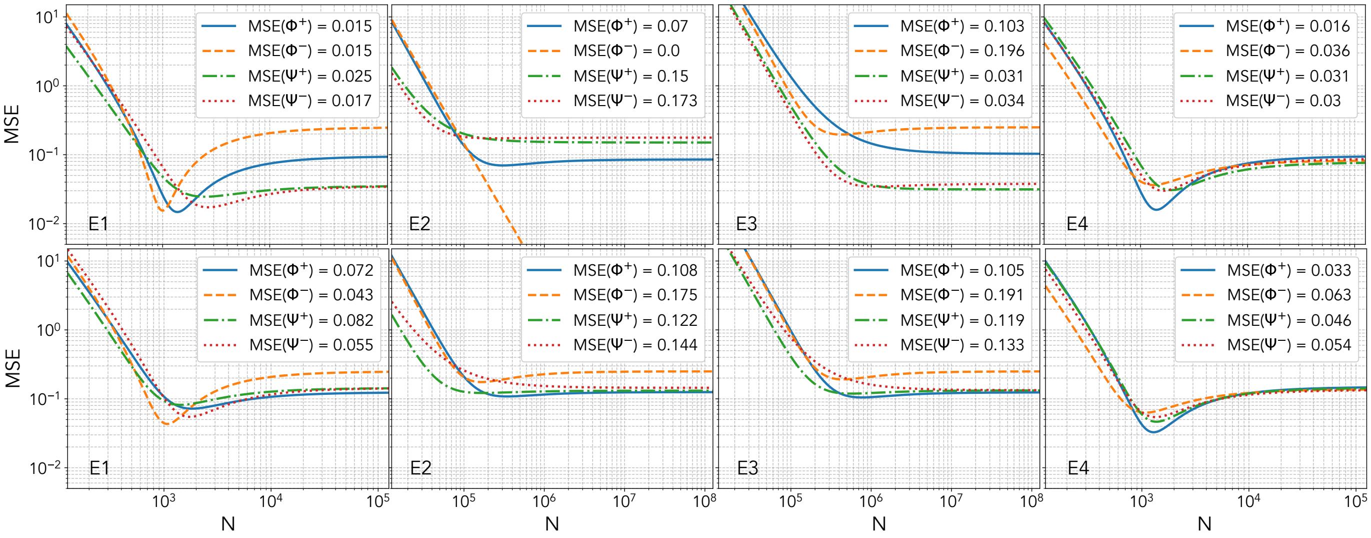

Figure S8. **Estimation errors with shadow tomography VS the input statistics.** MSEs for the configurations **E1**, **E2**, **E3**, **E4**, as a function of the unknown input statistics N, for separable (upper row) and entangled (bottom row) states, for the four Bell witnesses. In each case, large values of N correspond to the trivial estimator $b \mapsto \hat{o}(b) = 0$, corresponding to which the MSE approaches, for each target observable $\mathcal{O}$, the average of $\mathrm{Tr}(\mathcal{O}\rho)^2$ over the corresponding states. The observed linear behaviour of $\mathrm{MSE}(\Phi^-; N)$ in the upper **E2** plot has the same origin: in this configuration $\langle \mathcal{W}_{\Phi^-} \rangle = 0$ for all separable states, thus the zero estimator $b \mapsto \hat{o}(b)$ reproduces the correct value, and the MSE approaches it with the typical $1/N$ behaviour.

the true value of N. The MSE estimated with the true N could, therefore, be significantly higher than this lower bound. We nonetheless choose here to report the minimum, to avoid biasing our comparison in favor of the QELM. We find the shadow-tomography-based estimates to across the board yield MSEs significantly higher than those given via the QELM. For instance, in the **E1** configuration, for the witness $\mathcal{W}_{\Phi^+}$, we obtain via shadow tomography $\mathrm{MSE}(\mathcal{W}_{\Phi^+}) \approx 0.015$ for separable states and $\mathrm{MSE}(\mathcal{W}_{\Phi^+}) \approx 0.072$ for entangled states. In contrast, using the QELM trained solely on separable states, we achieve 0.002 for separable states and 0.041 for entangled states. When the QELM is trained on both separable and entangled states, the MSEs become 0.009 and 0.017, respectively. A brief summary of these numbers is given in table S3. Although these values will fluctuate across different experimental realizations, the enhanced performance of the QELM remains evident.

Method	MSE for separable states	MSE for entangled states
Shadow Tomography	0.015	0.072
QELM trained on separable	0.002	0.041
QELM trained on separable&entangled	0.009	0.017

Table S3. **QELM estimation benchmark.** Comparison of MSEs obtained from shadow tomography vs the QELM approach, for the **E1** configuration with target observable $\mathcal{W}_{\Phi^+}$.
